# Prediction of prostate cancer aggressiveness using ^18^F-Fluciclovine (FACBC) PET and multisequence multiparametric MRI

**DOI:** 10.1038/s41598-020-66255-8

**Published:** 2020-06-10

**Authors:** Parisa Movahedi, Harri Merisaari, Ileana Montoya Perez, Pekka Taimen, Jukka Kemppainen, Anna Kuisma, Olli Eskola, Jarmo Teuho, Jani Saunavaara, Marko Pesola, Esa Kähkönen, Otto Ettala, Timo Liimatainen, Tapio Pahikkala, Peter Boström, Hannu Aronen, Heikki Minn, Ivan Jambor

**Affiliations:** 1 Department of Future Technologies, University of Turku, Turku, Finland; 2 Department of Diagnostic Radiology, University of Turku, Turku, Finland; 3 Institute of Biomedicine, University of Turku and Department of Pathology, Turku University, Hospital, Turku, Finland; 4 Turku PET Centre, Turku University and Turku University Hospital, Turku, Finland; 5 Department of Clinical Physiology and Nuclear Medicine, Turku University Hospital, Turku, Finland; 6 Department of Oncology and Radiotherapy, Turku University Hospital, Turku, Finland; 7 Department of Medical Physics, Turku University Hospital, Turku, Finland; 8 Department of Urology, University of Turku and Turku University hospital, Turku, Finland; 9 A.I. Virtanen Institute for Molecular Sciences, University of Eastern Finland, Kuopio, Finland; 10 Research Unit of Medical Imaging, Physics and Technology, University of Oulu, Oulu, Finland; 11 Department of Clinical Radiology, Oulu University Hospital, Oulu, Finland; 12 Medical Imaging Centre of Southwest Finland, Turku University Hospital, Turku, Finland; 13 Department of Radiology, Icahn School of Medicine at Mount Sinai, New York, USA

**Keywords:** Urology, Prostate, Oncology, Cancer

## Abstract

The aim of this prospective single-institution clinical trial (NCT02002455) was to evaluate the potential of advanced post-processing methods for ^18^F-Fluciclovine PET and multisequence multiparametric MRI in the prediction of prostate cancer (PCa) aggressiveness, defined by Gleason Grade Group (GGG). 21 patients with PCa underwent PET/CT, PET/MRI and MRI before prostatectomy. DWI was post-processed using kurtosis (ADC_k_, K), mono- (ADC_m_), and biexponential functions (f, D_p_, D_f_) while Logan plots were used to calculate volume of distribution (V_T_). In total, 16 unique PET (V_T_, SUV) and MRI derived quantitative parameters were evaluated. Univariate and multivariate analysis were carried out to estimate the potential of the quantitative parameters and their combinations to predict GGG 1 vs >1, using logistic regression with a nested leave-pair out cross validation (LPOCV) scheme and recursive feature elimination technique applied for feature selection. The second order rotating frame imaging (RAFF), monoexponential and kurtosis derived parameters had LPOCV AUC in the range of 0.72 to 0.92 while the corresponding value for V_T_ was 0.85. _T_he best performance for GGG prediction was achieved by K parameter of kurtosis function followed by quantitative parameters based on DWI, RAFF and ^18^F-FACBC PET. No major improvement was achieved using parameter combinations with or without feature selection. Addition of ^18^F-FACBC PET derived parameters (V_T_, SUV) to DWI and RAFF derived parameters did not improve LPOCV AUC.

## Introduction

Prostate cancer (PCa) has wide range of aggressiveness, ranging from indolent disease to highly aggressiveness PCa^1,2^. Approximately 10–15% of men who undergo radical treatment (surgery or radiotherapy) for localized PCa will develop recurrence based on elevated blood levels of prostate-specific antigen (PSA)^3^. Radical treatment (surgery or radiotherapy) has side effects such as impotence and urinary incontinency^4,5^. Thus, accurate risk stratification of PCa is of utmost importance to both improve quality of life and PCa-specific survival. Prostate cancer aggressiveness is most commonly expressed as Gleason Grade Group (GGG), ranging from 1 to 5^6^. Unfortunately, GGG is commonly under-estimated based on systematic biopsy^7–9^. If improved PCa risk stratification could be achieved by non-invariance imaging methods, better patient tailored specific treatment options could lead to improved quality of life with decreased PCa specific mortality.

Multiple different magnetic resonance imaging (MRI) based methods have already been applied to estimate PCa aggressiveness non-invasively. Diffusion weighed imaging (DWI) has demonstrated potential to predict GGG of PCa^10–17^. Most commonly modelling of PCa DWI signal decay is performed using the monoexponential function^18^ which is the simplest mathematical model to characterize the signal decay. However, other models have been extensively applied for modelling of PCa DWI signal decay with mixed results^10–13,19,20^. Moreover, spin lock imaging methods^21^, such as relaxation along a fictitious field (RAFF)^22,23^, have been applied for estimation of PCa aggressiveness. Relaxation along a fictitious field method is performed under sub-adiabatic conditions and in prior studies was shown to predict cell density of glioma rat model^22,23^ and significantly correlated with GGG in human PCa^21,24^.

^18^F-fluorodeoxyglucose (FDG) positron emission tomography/computerized tomography (PET/CT) is widely used in oncology but it is rarely applied to evaluate patients with localized PCa due to low FDG uptake by PCa^25^. Anti-1-amino-3-[18 F]-fluorocyclobutane-1-carboxylic acid (^18^F-FACBC/Fluciclovine) is a promising technique for imaging of PCa and since the year 2016 approved by FDA in the setting of biochemical recurrence^26^. ^18^F-fluciclovine PET in addition with prostate-specific membrane antigen (PSMA) derivatives seems to be more sensitive than previously used PET tracers for early detection and characterization of PCa^27^.

The aim of the current study was to perform quantitative head-to-head comparison of multiple MRI and ^18^F-FACBC (Fluciclovine) PET methods for non-invasive GGG prediction of primary PCa.

## Results

Due to presence of inhomogeneous B_0_ field (static magnetic field) causing severe susceptibly artifacts, DWI and RAFF datasets of 5 patients were excluded from quantitative analysis of Gleason score prediction resulting into 21 patients and 36 tumors included. Representative imaging findings for these 5 patients are presented in the Supporting Material (FLUCIPRO_susceptibility_artifacts.pdf) while complete imaging data sets are presented at the study server - http://petiv.utu.fi/flucipro. Of these 36 tumors, 7, 13, and 16 had Gleason Grade Group 1 (Gleason score 3 + 3), 2 (Gleason score 3 + 4), >2 (Gleason score >3 + 4), respectively. Only 6 tumors (17%, 6/36) were located in central gland while the remaining tumors were in the peripheral zone. Patients’ characteristics are presented in Table 1. Median (IQR) PSA values was 6.2 (7.6–14.4) ng/ml.

**Table 1:** Patients’ characteristics.

Patient no	PSA	fPSA %	GS	LN removed	LN met+	3mo PSA
1	4.3	8.8	4 + 3 + 5	10	0	0.003
2	4.1	7.6	4 + 5	11	0	0.007
3	4.6	10	4 + 5	10	0	0.003
4	8.1	17.2	4 + 5	31	0	0.140
5	7.2	12.7	4 + 3/3 + 3	10	0	0.003
6	7.6	17.1	4 + 3	16	0	0.019
7	12	x	4 + 5/3 + 4	8	0	0.091
8	8.3	24.4	4 + 3/3 + 4	8	0	0.003
9	35	x	3 + 4/3 + 4	17	0	0.024
10	6.2	8.2	4 + 5/3 + 4/3 + 3 + 4	10	0	0.003
11	24	x	4 + 3	29	0	0.005
12	16	x	4 + 5	21	0	0.003
13	6.5	11.8	4 + 3 + 5/3 + 3	29	0	0.005
14	7.7	15.5	3 + 4/3 + 4/3 + 3	12	0	0.05
15	6.2	x	3 + 4/3 + 4	25	0	0.003
16	5.3	11	3 + 4/3 + 4/3 + 3	26	0	0.003
17	7.6	3.8	5 + 4/3 + 3	16	1	0.032
18	21	x	3 + 4/3 + 3	16	0	0.003
19	14	x	4 + 5/3 + 3	13	0	0.003
20	6.7	9.4	4 + 5	36	3	0.100
21	14.7	x	4 + 3	23	0	0.026

### Univariate analysis

**Figure 1:**
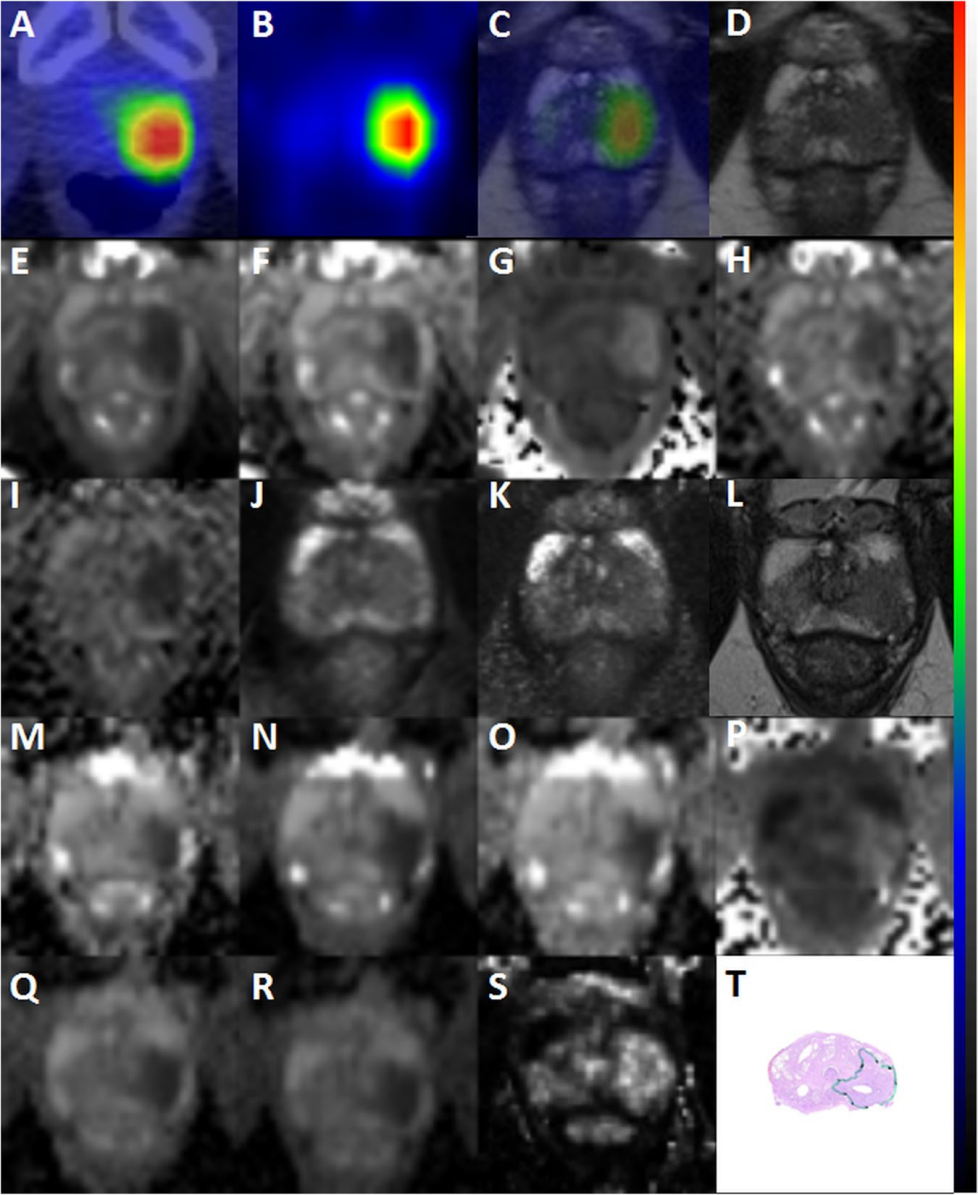
Focal uptake of ^18^F-FACBC (PET/CT - A, PET/MR - C) and increased distribution volume values (B) were present in the left lobe of patient no. 26 (65 years, 14.7 ng/ml, T2aN0). This area was associated with the following signal intensity changes: low signal intensity on T2-weighted weighted images (PET/MRI - D, mpMRI - L), decreased ADC_m_ values (ADC_m_0_2000_12b_ - E, ADC_m_0_500_14b_ - H, ADC_m_0_500_5b_ - M, ADC_m_0_2000_16b_ - N, ADC_m_0_1500_2b_ - Q, ADC_m_0_2000_2b_ - R), decreased D_f_ values (D_f_0_500_14b_ - I) decreased ADC_k_ values (ADC_k_0_2000_12b_ - F, ADC_m_0_2000_12b_ - O), increased K values (K__0_2000_12b_ - G, K__0_2000_16b_ - P), decreased T_RAFF_ values (J), decreased T_2_ (K), increased K^trans^ values (S). The corresponding whole mount prostatectomy section (T - tumor outline in green) demonstrates prostate cancer with Gleason score of 4 + 3. Images are scaled to as follows: SUV from 0 to 6.30 (A, C), volume of distribution 0 to 9 mL plasma/mL tissue (B), ADC_m_ 0 to 4.0 μm^2^/ms (E, H, M, N, Q, R), Df 0 to 4.0 μm^2^/ms (I), ADC_k_ 0 to 4.0 μm^2^/ms (F, O), K 0 to 3.0 (G, P), T_RAFF_ 0 to 300 ms, T_2_ 0 to 300 ms (K), K^trans^ 0 to 2.0 min^−1^ (S). Voxels are interpolated with trilinear interpolation.

**Figure 2:**
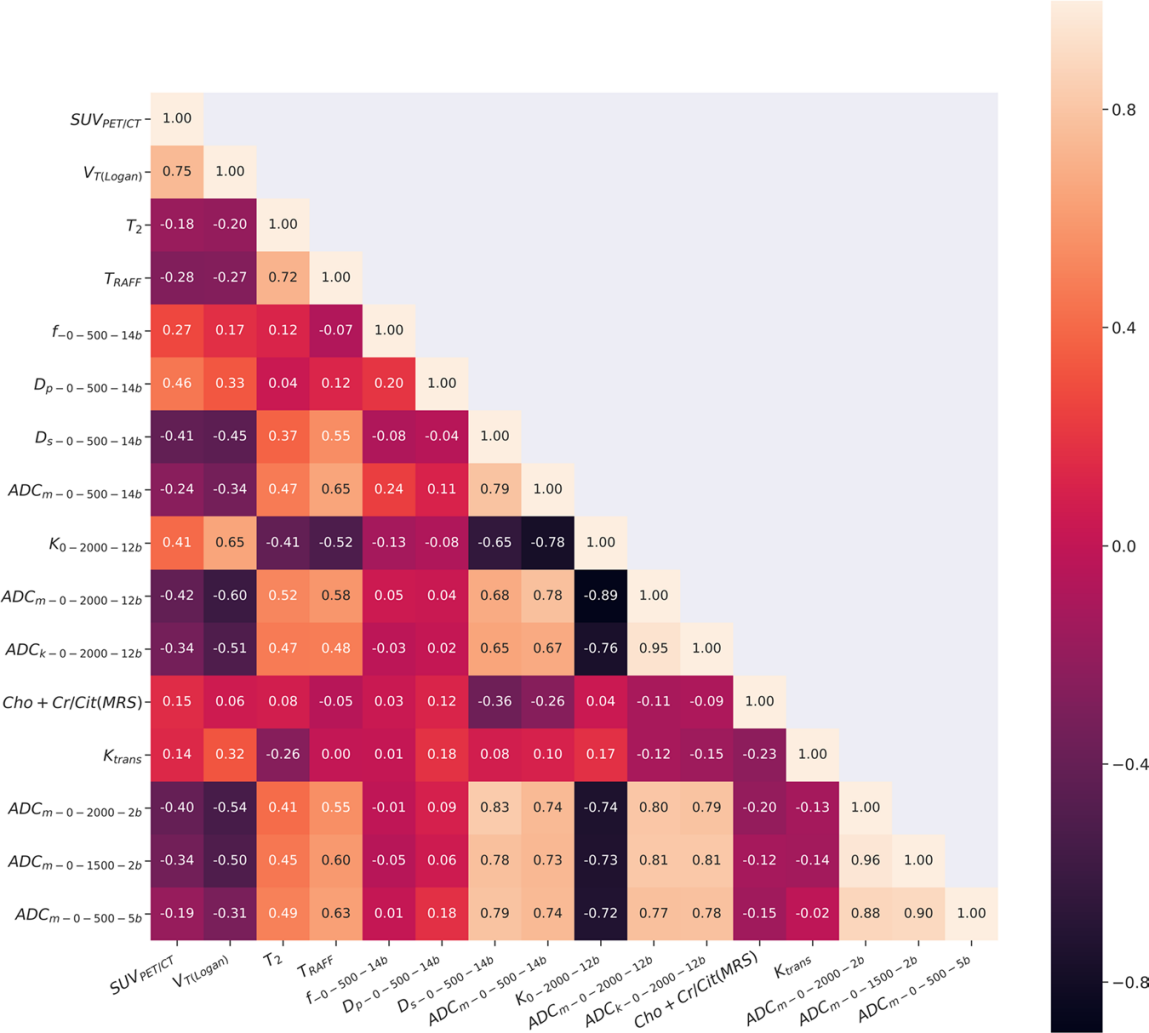
Spearman correlation coefficient matrix of all the 16 parameters: V_T (Logan)_, SUV_PET/CT_, T2, TRAFF, f__0_500_14b_, D_p _0_500_14b_, D_f_0_500_14b_, ADC_m_0_500_14b,_ ADC_k_0_2000_12b_, K__0_2000_12b_, ADC_m_0_2000_12b_, Cho+Cr/Cit (MRS), K^trans^, ADC_m_0_2000_2b_, ADC_m_0_1500_2b,_ ADC_m_0_500_5b_.

The AUC value of each variable and their 95% confidence interval together with the Spearman correlation coefficient and the corresponding p-values are presented in Table 2. The best performing features for PET/CT was V_T_ (from Logan plots) parameter which had significant positive correlation (p < 0.01) with GGG. The predicative power (as evaluated by AUC and ρ values) of PET/CT was similar for SUV values measure on frames between 12–22 minutes. Neither V_T_ values nor SUV of PET/CT demonstrated higher AUC or absolute ρ values than DWI derived parameters. All DWI derived parameter values had significant correlation (p < 0.05 or p < 0.01) with GGG except of f and D_p_ parameters (biexponential function [IVIM model] for low b-values DWI). The highest performance was achieved by K parameter of kurtosis model (Table 2). In addition to DWI derived parameters, T_RAFF_ values demonstrated significant correlation with Gleason score as well (Table 2). In contrast, T_2_, K^trans^ and Cho + Cr/Cit had low AUC values and non-significant ρ values. T_2_ mapping, ^1^H-MRS ((choline + creatine)/citrate)) and DCE-MRI (K^trans^) derived parameters had the lowest LPOCV AUC in the range of 0.35 to 0.67. Representative parametric maps are shown in Fig. 1. Spearman correlation coefficient between all individual parameters is shown in Fig. 2.

**Table 2:** Area under the curve values (AUC) for Gleason Grade Group classification of prostate cancer tumors and Spearman correlation coefficient values with Gleason Grade Groups.

Parameters	Scan time^#^	AUC (95% CI) GGG 1 (GS 3 + 3) vs GGG > 1 (>3 + 3)	Nested LPOCV AUC GGG 1 (GS 3 + 3) vs GGG > 1 (>3 + 3)	ρ (95% CI)
V_T (Logan)_	24 min	0.84 (0.70, 0.96)	0.84	0.49 (0.2 0.71)**
SUV_PET/CT_20–24min_	4 min	0.76 (0.57, 0.91)	0.76	0.37 (0.05 0.63)*
ADC_m_0_2000_12b_	8 min 48 s	0.83 (0.62, 0.98)	0.83	−0.48 (−0.70–0.17)**
ADC_k_0_2000_12b_	8 min 48 s	0.77 (0.54, 0.95)	0.77	−0.38 (−0.63–0.07)*
K__0_2000_12b_	8 min 48 s	0.92 (0.81, 1.0)	0.92	0.60 (0.34 0.78)**
ADC_m_0_500_14b_	3 min 45 s	0.83 (0.60, 1.0)	0.83	−0.47 (−0.70–0.17)**
f__0_500_14b_	3 min 45 s	0.51 (0.29, 0.73)	0.26	−0.02 (−0.35 0.31)
D_p _0_500_14b_	3 min 45 s	0.57 (0.30, 0.80)	0.31	0.09 (−0.24 0.41)
D_f_0_500_14b_	3 min 45 s	0.82 (0.56, 0.99)	0.82	−0.46 (−0.68–0.16)**
T_RAFF_	5 min 51 s	0.82 (0.61, 0.97)	0.82	−0.46 (−0.69–0.16)**
T_2_	1 min 35 s	0.67 (0.38, 0.92)	0.67	−0.24 (−0.53 0.10)
ADC_m_0_500_5b_	5 min 06 s	0.89 (0.70, 1.0)	0.89	−0.56 (−0.75–0.29)**
ADC_m_0_1500_2b_	1 min 37 s	0.85 (0.65, 0.99)	0.85	−0.51 (−0.72–0.22)**
ADC_m_0_2000_2b_	1 min 37 s	0.86 (0.65, 1.0)	0.86	−0.52 (−0.73–0.25)**
K_trans_	7 min 28 s	0.63 (0.41, 0.85)	0.63	0.19 (−0.15 0.49)
Cho+Cr/Cit (MRS)	12 min 36 s	0.53 (0.30,0.75)	0.35	0.04 (−0.30 0.36)

GGG = Gleason Grade Group; GS = Gleason score, LPOCV AUC = nested leave-pair out cross validation area under the curve; CI = confidence interval; ρ - Spearman correlation coefficient; *p value < 0.05; **p value < 0.01; # - Scan time does not include shimming and calibration.; SUVPET/CT_20–24min is the maximum standardized uptake value of averaged the last time frame (20–24 min).

### Multivariate analyses

The prediction performance of the logistic regression model of all the 16 parameters together (features) had the LPOCV AUC of 0.84 (Table 3). The combinations of individual features with their corresponding sequence times are shown in Table 3. The overall sequence time for all data acquisition was 72 minutes and 23 seconds (Table 3). However, the actual time to collect all data was longer since shimming and calibrations are not included in this time. No major improvement was achieved using different number of parameter combinations (Table 3).

**Table 3:** Area under the curve values (AUC) of multivariate analysis of the different combinations of parameters.

Scan time^#^	Parameters	Nested LPOCV AUC GGG 1 (GS 3 + 3) vs GGG > 1 (>3 + 3)
72 min 23 s	V_T (Logan),_ SUV_PET/CT,_ T_2_, T_RAFF_, f__0_500_14b_, D_p _0_500_14b_, D_f_0_500_14b_, ADC_m_0_500_14b_, ADC_k_0_2000_12b_, K__0_2000_12b_, ADC_m_0_2000_12b_ Cho+Cr/Cit (MRS), K^trans^, ADC_m_0_2000_2b_ ADC_m_0_1500_2b,_ ADC_m_0_500_5b_	0.84
14 min 39 s	ADC_m_0_2000_12b,_ K__0_2000_12b,_ T_RAFF_	0.89
16 min 14 s	ADC_m_0_2000_12b,_ K__0_2000_12b,_ T_RAFF,_ T_2_	0.93
8 min 20 s	ADC_m_0_2000_2b_ ADC_m_0_1500_2b,_ ADC_m_0_500_5b_	0.86
17 min 42 s	ADC_m_0_500_5b,_ K^tran^, Cho+Cr/Cit (MRS)	0.81
8 min 48 s	ADC_m_0_2000_12b,_ K__0_2000_12b_	0.90
32 min 48 s	ADC_m_0_2000_12b,_ K__0_2000_12b,_ V_T (Logan)_	0.89
38 min 39 s	ADC_m_0_2000_12b,_ K__0_2000_12b,_ V_T (Logan),_ T_RAFF_	0.90
25 min 37 s	ADC_m_0_2000_12b,_ V_T (Logan)_	0.86
24 min 0 s	V_T (Logan),_ SUV_PET/CT_20–24min_	0.82
29 min 6 s	ADC_m_0_500_5b,_ V_T (Logan)_	0.94

GGG = Gleason Grade group, GS = Gleason score; LPOCV AUC = nested leave-pair out cross validation area under the curve; CI = confidence interval; ρ - Spearman correlation coefficient; # - Scan time does not include shimming and calibration.; SUVPET/CT_20–24min is the maximum standardized uptake value of the last time frame (20–24 min).

Furthermore, all 16 quantitative parameters were grouped into four imaging groups: routine MRI, ^18^F-FACBC (Fluciclovine) PET, DCE-MRI and RAFF. Using recursive feature elimination technique for routine MRI group with 12 parameters, two DWI parameters (ADCm_0_500_5b and K_0_2000_12b) were selected and demonstrated LPOCV AUC of 0.86 (Table 4). No major improvements were achieved using parameter combinations of the four imaging groups, without (Table 4) or with feature selection (Table 5) compared with univariate analyses (Table 2).

**Table 4:** Classification performance of parameters based on four imaging group (routine MRI, ^18^F-Fluciclovine PET, DCE-MRI and RAFF) without any feature selection.

Parameters	Nested LPOCV AUC
	GGG 1 (GS 3 + 3) vs GGG > 1 (>3 + 3)
**Routine MRI**:	0.74
T_2,_ f__0_500_14b_, D_p _0_500_14b_, D_f_0_500_14b_, ADC_m_0_500_14b_, ADC_k_0_2000_12b_, K__0_2000_12b_, ADC_m_0_2000_12b_ Cho+Cr/Cit (^1^H-MRS), ADC_m_0_2000_2b_ ADC_m_0_1500_2b,_ ADC_m_0_500_5b_	
^**18**^**F-Fluciclovine PET:**	0.82
V_T (Logan),_ SUV_PET/CT_20–24min_	
**DCE-MRI:**	0.63
K_trans_	
**RAFF:**	0.82
T_RAFF_	
**Routine MRI** + ^**18**^**F-Fluciclovine PET**	0.83
**Routine MRI** + **DCE-MRI**	0.79
**Routine MRI** + **RAFF**	0.80
^**18**^**F-Fluciclovine PET PET** + **DCE-MRI**	0.78
^**18**^**F-Fluciclovine PET** + **RAFF**	0.88
**DCE-MRI** + **RAFF**	0.80

LPOCV AUC = nested leave-pair out cross validation area under the curve; GGG = Gleason Grade group; GS = Gleason score.

**Table 5:** Feature selection for each combination of the imaging groups (routine MRI, ^18^F-Fluciclovine PET, DCE-MRI and RAFF). Best predictive features are stated in selected feature section for each of the 4 imaging groups and their combinations.

Parameters	AUC	Selected features
	GGG 1 (GS 3 + 3) vs GGG > 1 (>3 + 3)	
**Routine MRI**:	0.86	ADC_m_0_500_5b,_ K__0_2000_12b_
T_2,_ f__0_500_14b_, D_p _0_500_14b_, D_f_0_500_14b_, ADC_m_0_500_14b_, ADC_k_0_2000_12b_, K__0_2000_12b_, ADC_m_0_2000_12b_ Cho+Cr/Cit (MRS), ADC_m_0_2000_2b_ ADC_m_0_1500_2b,_ ADC_m_0_500_5b_		
**Routine MRI** + ^**18**^**F-Fluciclovine PET**	0.86	ADC_m_0_500_5b,_, V_T (Logan)_
**Routine MRI** + **DCE-MRI**	0.86	ADC_m_0_500_5b,_ K__0_2000_12b_
**Routine MRI** + **RAFF**	0.87	ADC_m_0_500_5b,_ K__0_2000_12b_

LPOCV AUC = nested leave-pair out cross validation area under the curve; GGG = Gleason Grade.

group, GS = Gleason score.

## Discussion

Prostate cancer remains a leading cause of cancer-related death in the western world^1^ with wide range aggressiveness. Accurate risk stratification of men with suspected or diagnosed PCa is of utmost importance. In this analysis of prospective single institutional clinical trial (ClinicalTrials.gov Identifier: NCT02002455), we have evaluated 16 unique quantitative parameters derived from ^18^F-Fluciclovine (FACBC) PET and multisequence multiparametric MRI. ^18^F-FACBC (Fluciclovine) PET, RAFF, DWI (monoexponential and kurtosis derived parameters) demonstrated good correlation and classification performance (GGG 1 vs >1) for GGG of PCa tumors based on whole mount prostatectomy sections as reference standard. In contrast, T2-mapping, ^1^H-MRS ((choline + creatine)/citrate)) and DCE-MRI (K^trans^) derived parameters failed to predict GGG. No major improvement was achieved using parameter combinations with or without feature selection feature.

Multiple previous studies evaluated performance of ^18^F-Fluciclovine (FACBC) PET/CT in men with primary PCa. Suzuki *et al*.^28^ in a study of 47 patients who underwent radical prostatectomy following ^18^F-Fluciclovine (FACBC) PET/CT reported sensitivity and specificity of 92.5% (173/187 segments) and 90.1% (64/71 segments), respectively. However, predictive performance of quantitative parameters derived from ^18^F-Fluciclovine (FACBC) PET/CT for GGG/Gleason score prediction was not explored^28^. Turkbey *et al*.^29^ in a study of 21 patients who underwent radical prostatectomy following ^18^F-Fluciclovine (FACBC) PET/CT showed that SUVmax from ^18^F-Fluciclovine (FACBC) PET/CT were significantly higher in the tumor than in the normal prostate (4.5 ± 0.5 vs 2.7 ± 0.5, respectively, p < 0.001) but with overlap in areas with benign prostate hyperplasia (4.5 ± 0.5 vs 4.3 ± 0.6, respectively). SUVmax was correlated with Gleason score and demonstrated Spearman rank correlation of 0.53. However, comparison of quantitative parameters derived from ^18^F-Fluciclovine (FACBC) PET/CT with MRI quantitative parameters was not performed. In contrast to Turkbey *et al*.^29^, Schuster *et al*.^30^ found considerable SUVmax overlap between PCa and benign tissue in 10 patients with PCa. We have reported on details of ^18^F-Fluciclovine (FACBC) PET/CT reported both qualitative and quantitative evaluation in 26 patients with PCa elsewhere^31^. The current study focuses only on quantitative parameters derived from ^18^F-Fluciclovine (FACBC) PET/CT, PET/MRI and multiparametric MRI. It is the first head-to-head comparison of multiple different DWI methods vs spin locking vs T_2_-mapping vs ^18^F-Fluciclovine (FACBC) PET. In total, five different DWI acquisitions were collected, two with 3 T Ingenuity TF PET/MRI scanner and three with 3 T Magnetom Verio scanners in addition to spin locking (RAFF), T2 mapping, FACBC PET (24 min scan), ^1^H-MRS and DCE-MRI.

Magnetic Resonance Imaging has been successfully integrated in in the diagnostic pathways for PCa. Imaging methods such as MRI or PET gives an opportunity to localize and potentially characterize PCa lesions and to eventually diagnose or treat any suspicious lesions in contrast to other tools such as biomarkers. Multiple prospective single (eg. BIDOC^32^, IMPROD^33^) and multi-institution trials (eg. PROMIS^34^, PRECISION^35^, MULTI-IMPROD^36^, MRI-FIRST^37^, 4M^38^) demonstrated the potential of prostate MRI to limit number of unnecessary biopsies in men with suspected PCa. PET imaging is currently not recommended to be performed in men with suspected or diagnosed PCa. Our findings does not support the use of ^18^F-Fluciclovine (FACBC) PET in men with suspected or diagnosed PCa since a) added value of visually reported ^18^F-Fluciclovine (FACBC) PET to high quality prostate MRI was shown to be limited^31^, b. quantitative parameters derived from ^18^F-Fluciclovine (FACBC) PET/CT did not outperform high quality DWI parameters for Gleason score prediction, c. accuracy of ^18^F-Fluciclovine (FACBC) PET for lymph node detection appears to be limited^31^.

Diffusion weighed imaging has been extensively applied for PCa detection and characterization. Large number of prior studies demonstrated correlation of ADC_m_, quantitative DWI parameter obtained using monoexponential function, with Gleason score. Prior studies reported spearman correlation coefficient (ρ values) values in the range from −0.30/−0.36 to −0.60^10–17^. In contract to prior studies, five different DWI acquisitions were collected in the current study, two with 3 T Ingenuity TF PET/MRI scanner and three with 3 T Magnetom Verio scanner. In the current study DWI (monoexponential and kurtosis function derived parameters) demonstrated good correlation with Gleason score/GGG and PCa classification performance (GGG 1 vs >1) in the current study. Large proportion (78%, 28/36) of PCa patients had GGG > 1. This fact as well as differences in DWI data quality and post-processing could explain higher AUC and ρ values for PCa detection and characterization in the current study compared with previous reports. In contrast to monoexponential and kurtosis function, biexponential intravoxel incoherent motion imaging derived parameters (f, D_p_) failed to predict GGG similar to T_2_-mapping, ^1^H-MRS ((choline + creatine)/citrate)) and DCE-MRI (K^trans^).

In the current study both univariate and multivariate analysis with AUC as the evaluation criteria was used to evaluate the potential of the fitted parameters to correctly classify tumors into GGG. Moreover, ρ values were used to estimate correlation of quantitative parameters with GGG. Gleason Grade Group based on systematic^7–9^, and to lesser degree on MRI-targeted^39^, biopsy commonly underestimates true GGG detected in prostatectomy specimens. Since GGG differ between biopsy and prostatectomy, whole mounts prostatectomy sections are ideal for validation of an imaging technique to detect PCa and predict its potential. However, using GGG/Gleason score based on prostatectomy samples introduces inclusion bias because only men with PCa who underwent prostatectomy can be included.

Our study has multiple limitations. First of all, the study is limited by relatively small number of PCa lesions. Only 6 tumors (17%, 6/36) were located in the central gland, and thus, a separate analysis for central gland tumors was not performed. It is well known that correlation of whole mount prostatectomy section to *in vivo* imaging data sets can be challenging. In the current study, all of ROIs were placed by one research fellow (IJ) working in collaboration with pathologist (PT). Thus, inter-reader variability has not been evaluated. Furthermore, mean signal intensity per ROIs was fitted in contrast to the use of statistical measures (eg. mean, median, percentile, kurtosis, etc …) from voxel-by-voxels fits. Due to use of mean signal per ROI the information concerning tumor and tissue heterogeneity has not been utilized. Development of fully automatic tools for ROI positioning and data extraction from PET/MRI is needed to allow high reproducibility of results in multi-reader setting.

In conclusion, we have shown that some of the ^18^F-FACBC (Fluciclovine) PET and MRI derived parameters demonstrated potential to predict PCa aggressiveness estimated by GGG. In total, 16 unique PET (V_T_, SUV) and MRI derived quantitative parameters were evaluated. DWI parameter (K of kurtosis function) had the highest AUC and ρ values for GGG prediction. No major improvements were achieved using parameter combinations with or without feature selection.

## Methods

Between January 2014 and June 2015, 32 patients with histologically confirmed PCa were prospectively enrolled in a single center clinical trial (ClinicalTrials.gov Identifier: NCT02002455). Following enrolment, 26 patients underwent ^18^F-FACBC PET/CT followed by ^18^F-FACBC PET/MRI performed in succession and mpMRI performed within 7 days. The trial was conducted according to Declaration of Helsinki regulation and each patient gave written informed consent before enrolment. The trial was approved by institutional review board of the Turku University Hospital, Turku, Finland.

### PET/CT

PET/CT was performed immediately after injection of 369 ± 10 of 18F-FACBC (Fluciclovine) MBq PET/CT using a combined Discovery 690 PET/CT scanner (General Electric Medical Systems, Milwaukee, US) as detailed previously^31^. Dynamic data collection using a list-mode acquisition was performed for 20 minutes with additional table positions covering the whole pelvis and abdomen collected for a 4-min duration per table position. The dynamic data were reconstructed to five frames with a frame time of 4 min. The PET data was reconstructed with all quantitative corrections applied including detector dead time, decay, randoms, scatter, and photon attenuation. PET images were reconstructed in a 128 × 128 matrix with a voxel size of 5.47 × 5.47 × 3.27 mm^3^, using the VUE Point FX algorithm with time-of-flight technology and a 6-mm Gaussian post-filter and no resolution modeling.

### PET/MRI

Immediately following completion of PET/CT imaging, each patient was transferred to the Ingenuity TF PET/MRI scanner (Phillips Medical Systems, Cleveland, Ohio, US) as detailed previously^31^. Manufacturer’s surface array 32-channel cardiac coil was used as a received coil, no endorectal coil was used. MR-based attenuation correction (MRAC) was performed using the vendor-provided method with Repetition Time/Echo Time (TR/TE) 4.0/2.3 ms and flip angle 10°. The PET/MRI studies were started with the MRAC in the MRI gantry, followed by patient’s table moved to PET gantry for PET imaging. Two table positions each of 4 minutes duration and covering the whole pelvis were acquired. PET image reconstructions were performed with all quantitative corrections, taking into account detector dead time, decay, randoms, scatter, and photon attenuation. PET images were reconstructed in a 144 × 144 matrix with an isotropic voxel size of 4 mm using a blob-OSEM algorithm with time-of-flight.

Following PET data collection, patient’s table was moved back to the MR scanner gantry for MRI data acquisition and performed as previously described^31^. T2-weighted images were acquired using a single shot TSE sequence with TR/TE 4668/130 ms, FOV 252 × 320 mm^2^, acquisition matrix size 250 × 320, reconstruction matrix size 512 × 672, slice thickness 2.5 mm, partial Fourier factor of 0.6, SENSE^40^ factor of 2, and sequence time 1 minute 10 seconds. Second order rotating frame (RAFF) has been used in the current study^41^ with the pulse train durations of 0, 45, and 90 ms and 500 Hz (γB_1_/2π) RF peak amplitude which corresponds to 11.74 µT (B_1_). RAFF acquisition was performed acquired using 3D T1-FFE sequence as a readout with the following parameters: TR/TE 4.0/2.3 ms; FOV 250 × 250 mm^2^; acquisition matrix size 168 × 144; reconstruction matrix size 384 × 384; slice thickness 5.0 mm; number of slices 6; flip angle 20; TFE factor 10; centric k-space coding; partial Fourier factor 0.625; SENSE^40^ factor 2; RAFF pulse interval 3000 ms and sequence time 5 minutes and 51 s. Two separated diffusion weighed imaging (DWI) acquisitions were performed. Both DWI data sets were acquired using a single shot spin-echo based sequence with monopolar diffusion gradient scheme and echo-planar read out in two separate acquisitions consisting of 14 (low b value set) and 12 (high b value set) b-values. The low b-value set was obtained using the following parameters: TR/TE 1394/44 ms, FOV 250 × 250 mm^2^, acquisition matrix size 100 × 99, reconstruction matrix size 224 × 224, slice thickness 5.0 mm, diffusion gradient timing 21.204 ms, diffusion gradient duration 6.6 ms, b-values 0, 2, 4, 6, 9, 12, 14, 18, 23, 28, 50, 100, 300, 500 s/mm^2^, sequence time 3 minutes 45 seconds. The high b-value set was acquired using the following parameters: TR/TE 3141/51 ms, FOV 250 × 250 mm^2^, acquisition matrix size 124 × 124, reconstruction matrix size 256 × 256, slice thickness 5.0 mm, diffusion gradient timing 24.5 ms, diffusion gradient duration 12.6 ms, b values 0, 100, 300, 500, 700, 900, 1100, 1300, 1500, 1700, 1900, 2000 s/mm^2^, sequence time 8 minutes 48 seconds. T2 relaxation values were measured using GraSE sequence with TR/TEs of 686/20, 40, 60, 80,100 ms, FOV 230 × 183 mm^2^, acquisition matrix size 256 × 163, reconstruction matrix size 512 × 400, slice thickness 5.0 mm, sequence time 1 minutes 35 seconds. The total duration of the PET/MRI examination was about 60 minutes. Detailed importable protocol (FLUCIPRO_PET_MRI.ExamCard) is available up request.

### Multiparametric MRI

Multiparametric MRI was performed using a 3 T MR scanner (Magnetom Verio 3 T, Siemens Healthcare, Erlangen, Germany) and surface array coils, no endorectal coil was used. The imaging consisted of triplanar T2-weighted (T2w) turbo spin-echo imaging, single shot spin-echo based DWI (three separate acquisitions), three-dimensional ^1^H-MRS and DCE-MRI^42^. Anatomical tri-planar T2-weighted images were acquired using a turbo spin-echo sequence with TR/TE 6400–8640/101 ms, FOV 200 × 200 mm^2^, reconstruction matrix size 320 × 320, slice thickness 3 mm, number of signal averages 2 and sequence time 2 minutes 17 seconds (transverse images), 2 minutes 28 seconds (sagittal images), 2 minutes 47 seconds (coronal images). Single shot spin-echo based DWI was performed using three separate acquisitions using the following b values: 1. 5 b values^42^: 0, 100, 200 350, 500 s/mm², sequence time 5 minutes 6 seconds; 2. 2 b-values^33^: 0, 1500 s/mm², sequence time 1 minute 51 seconds; 3. 2 b-values^33^: 0, 2000 s/mm², sequence time 1 minute 51 seconds. Additional parameters for DWI performed with 5 b-values are as follows: TR/TE 5543/80 ms, FOV 260 × 260 mm^2^, reconstruction matrix size 128 × 128, slice thickness 3 mm, number of signal averages 4, a bandwidth 1184 Hz/pixel acquiring 6/8 (75%) of k-space in phase-encoding direction, b-values 0, 100, 200 350, 500 s/mm², diffusion gradients in three orthogonal directions for each b-value, GRAPPA^43^ with the acceleration factor of 2 and 32 reference lines. DWI acquisitions performed using 2 b values of 0, 1500 s/mm², and separate 2 b values of 0, 2000 s/mm² were obtained using slice thickness of 5 mm (versus 3.0 mm for DWI with b-values 0, 100, 200 350, 500 s/mm²) to compensate for signal-to-noise ratio loss. The three-dimensional ^1^H-MRS covering whole prostate was performed using point resolved spatially localized spectroscopy (PRESS) sequence. Automatic and manual shimming of whole prostate volume was performed (to optimize the main magnetic field homogeneity) in every patient. Weighted averaging (NSA: 6) of elliptically sampled k-space, Hanning filtering of the signal and zero-filling to a 16 × 16 matrix was performed before Fourier transformation^44^. The TR and TE were optimized for the shape of citrate resonance^45^. Additional water and lipid signal from adjacent tissues was suppressed with seven outer volume saturation slabs. The following additional imaging parameters were used: acquisition bandwidth 1300 Hz, 512 spectra data points, FOV 96 × 96 × 96 mm^2^ and matrix size of 12 × 12 × 12 resulting into nominal voxel size of 8 × 8 × 8 mm^3^. A real voxel size could be best approximated as a sphere with a volume of 1.51 cm^3^ and diameter of 14.24 mm after apodization. The sequence time was 12 minutes 36 seconds while the actual acquisition time was about 15–20 minutes including manual shimming. Finally, axial DCE-MRI was performed before, during and after injection of contrast agent. Contrast agent (0.1 mmol/kg Dotarem, Guerbet, France) was injected 30 seconds after the beginning of the sequence through a peripheral vein at a rate of 2 ml/s via a mechanical injector (Spectris, Medrad, Indianola, USA). In total, 60 time points at a temporal resolution of 6.9 seconds were acquired using a three-dimensional VIBE sequence^46^ with the following parameters: TR/TE 5.43/1.87 seconds, 15 degree flip angle, FOV 240 × 240 mm^2^, reconstruction matrix size 192 × 192, slice thickness 3 mm, a bandwidth of 260 Hz/pixel acquiring 6/8 (75%) of k-space in phase-encoding direction, GRAPPA with the acceleration factor of 2 and 24 reference lines, acquisition time 6 minutes 54 seconds. Before the contrast enhanced MR imaging, images with five different flip angles of 2, 5, 8, 10, 15 degrees were obtained for calculation of pre-contrast longitudinal relaxation time (T10)^47^. The total duration of the MRI examination was about 70 minutes. Detailed importable protocol (FLUCIPRO_mpMRI.pdf) is provided in the Supporting Material.

In total, five different DWI acquisitions were collected, two with Ingenuity TF PET/MRI scanner and three with 3 T Magnetom Verio scanner (To differentiate DWI acquisitions, each DWI acquisition is assigned b value range and number of b values eg. DWI_0_2000_12b - DWI data collected using 12 b-values in the range of 0 to 2000 s/mm^2^): 3 T Ingenuity TF PET/MRI scanner: DWI_0_2000_12b, DWI_0_500_14b; 3 T Magnetom Verio scanner: DWI_0_500_5b, DWI_0_1500_2b, DWI_0_2000_2b.

### Quantitative modeling

Regions of interest for PET/CT, PET/MRI and multiparametric MRI data sets were placed in center of the PCa lesions using whole mount prostatectomy sections as reference standard. For PET/CT, maximum standardized uptake value of ROI voxel (SUV) were calculated according to the formula: SUV = [tissue radioactivity concentration (Bq/ml) × body weight(g)]/injected dose(Bq). The maximum standardized uptake value of the last time frame (20–24 min) was used in the current analysis. The data sets were analyzed using Advantage Workstation (version4.4, General Electric Medical Systems, Milwaukee, WI, USA). Logan plots with reference region placed in iliac/femoral artery were used to estimate the distribution volume (V_t_) of the tracer based on the assumption that transport of ^18^F-FACBC into cells is similar to receptor binding kinetics^48^.

DWI data sets were post-processed using kurtosis, mono-, and biexponential functions using validated fitting algorithm which are publicly available^19,20^.

The following models were applied to DWI data sets^19,20^:

1. Monoexponential model^18^:

1 $$S(b)={S}_{0}{e}^{-bAD{C}_{m}}$$

2. Biexponential model for low b-values DWI data set^49^:

2 $$S(b)={S}_{0}(f{e}^{-b{D}_{m}}+(1-f){e}^{-b{D}_{f}}$$

4. Kurtosis model for high b-values DWI data set^50^:

3 $$S(b)={S}_{0}{e}^{\left(-,b,A,D,{C}_{k},+,\frac{1}{6},{b}^{2},A,D,{C}_{k}^{2},K\right)}$$

The estimated parameters were: ADC_m_ is the diffusion coefficient of the monoexponential model, D_p_ is the intravoxel incoherent motion component of IVIM model (“pseudo-diffusion”), f is the proportion of “pseudo-diffusion”, D_f_ is the “fast” diffusion component, ADC_k_ is the diffusion coefficient of the kurtosis model, and K is the kurtosis. To differentiate parameters estimated using different data sets, each parameter is assigned b value range and number of b values eg. ADC_m_0_2000_12b_ − ADC_m_ of DWI data collected using 12 b-values in the range of 0 to 2000 s/mm^2^.

Two parameter monoexponential function was used to estimated RAFF (T_RAFF_) and T_2_ relaxation values.

In total, 16 unique features, two PET (V_t_, SUV_max_ of PET/CT) and 14 MRI derived, quantitative parameters were evaluated (Table 1). SUV of PET/MRI was not considered due to low diagnostic performance related to late post-injection time acquisition.

### Univariate analysis

A univariate analysis based on Area under the curve (AUC) values were used to estimate the potential of the quantitative parameters to predict Gleason grade group 1 vs >1 (Gleason score 3 + 3 vs >3 + 3). For each of the 16 parameters from the imaging modalities, area under the curve (AUC) values for PCa detection between the quantitative parameters and Gleason Grade Groups were determined along with 95% confidence intervals calculated using 2,000 bootstrap samples. Spearman correlation coefficient with the significance value and confidence intervals was calculated to assess the correlation between each parameter and the Gleason scores.

### Multivariate analysis

The regularized logistic regression based classifier from python scikit-learn library^51^ was used to assess the performance of the different combinations of quantitative parameters. L2-regularization was applied to avoid overfitting the data^52^. The predictive performance of the built models was estimated by a nested cross validation strategy, which consisted of an outer leave-pair-out cross-validation (LPOCV)^53^ for model performance evaluation and an inner leave-one-out cross validation (LOOCV) for hyper-parameter selection. In the LPOCV every possible pair of data points were held out at a time as test set, while the remaining data formed the training set used to build the model for predicting on the held out pair. Area under the receiver operating characteristic curve (AUC) was used to evaluate performance of each model. Moreover, a feature selection with nested cross-validation scheme was built to select the best subset of predictive quantitative parameters. The outer loop logistic regression with l2 norm and regularization parameter 1 was used to estimate the model performance based on the test data. The inner loop with Recursive feature elimination technique (RFE-CV) was used to choose the best subset of features based on train data^51,54^. Feature ranking was done with recursive feature elimination and a leave-one-out cross-validation was used for the selection of the best number of features. Finally, Spearman correlation coefficient analysis was conducted to evaluate the cross-correlation between the parameters.

### Histopathological analysis

The whole mount prostatectomy sections were prepared following robot assisted laparoscopic prostatectomy. The hematoxylin-eosin stained histological slides were first reviewed by one staff board certified pathologist and later re-reviewed by one experienced genitourinary pathologist. In the cases there were differences between the two reviews, the opinion of the third pathologist (senior genitourinary pathologist) was asked and consensus was reached between the genitourinary pathologists. The histology slice thickness was approximately 4–5 mm. Gleason Grade Group were assigned to tumors as combinations of primary, secondary, and tertiary Gleason grade, as defined by International Society of Urological Pathology (ISUP) guidelines^55^.

## Supplementary information

Supplementary information.

Supplementary information2.

Supplementary information3.

## Data Availability

Free public access to all imaging data sets, scanned whole mount prostatectomy sections and all variables used in the current manuscript is provided at the following address: http://petiv.utu.fi/flucipro Access to all variables used in the current manuscript is provided in Supporting Table S1 (Supporting_Table S1_FLUCIPRO_All_used_Variables.xlxs).
