# Supplementary material for: Prediction of prostate cancer aggressiveness using 18F-Fluciclovine (FACBC) PET and multisequence multiparametric MRI
Source: Sci Rep. 2020 Jun 10;10:9407. doi: 10.1038/s41598-020-66255-8 (PMC7287051; doi:10.1038/s41598-020-66255-8)

Prediction of prostate cancer aggressiveness using ^{18}F-Fluciclovine (FACBC) PET and multisequence multiparametric MRI

Parisa Movahedi[1,2], Harri Merisaari[1,2], Ileana Montoya Perez[1,2], Pekka Taimen[3], Jukka Kemppainen[4,5], Anna Kuisma[6], Olli Eskola[4], Jarmo Teuho[4], Jani Saunavaara[7], Marko Pesola[2], Esa Kähkönen[8], Otto Ettala[8], Timo Liimatainen[9,10,11], Tapio Pahikkala[1], Peter Boström[8], Hannu Aronen[2,12], Heikki Minn[4,6], Ivan Jambor[2,13]

[1] Department of Future Technologies, University of Turku, Turku, Finland

[2] Department of Diagnostic Radiology, University of Turku, Turku, Finland

[3] Institute of Biomedicine, University of Turku and Department of Pathology, Turku University Hospital, Turku, Finland

[4] Turku PET Centre, Turku University and Turku University Hospital, Turku, Finland, Finland

[5] Department of Clinical Physiology and Nuclear Medicine, Turku University Hospital, Turku, Finland

[6] Department of Oncology and Radiotherapy, Turku University Hospital, Turku, Finland

[7] Department of Medical Physics, Turku University Hospital, Turku, Finland

[8] Department of Urology, University of Turku and Turku University hospital, Turku, Finland

[9] A.I.Virtanen Institute for Molecular Sciences, University of Eastern Finland, Kuopio, Finland

[10] Research Unit of Medical Imaging, Physics and Technology, University of Oulu, Oulu, Finland

[11] Department of Clinical Radiology, Oulu University Hospital, Oulu Finland

[12] Medical Imaging Centre of Southwest Finland, Turku University Hospital, Turku, Finland

[13] Department of Radiology, Icahn School of Medicine at Mount Sinai, New York, USA

Supporting Material

Imaging data sets of 5 patients were excluded from quantitative analysis of Gleason score prediction due to presence of inhomogeneous B0 field (static magnetic field) causing severe susceptibly artifacts. Imaging findings of these 5 patients are presented below:

TSE = Turbo Spin Echo

Ssh SE EPI = Single Shot Spin Echo Echo-Planar read-out

No. 1

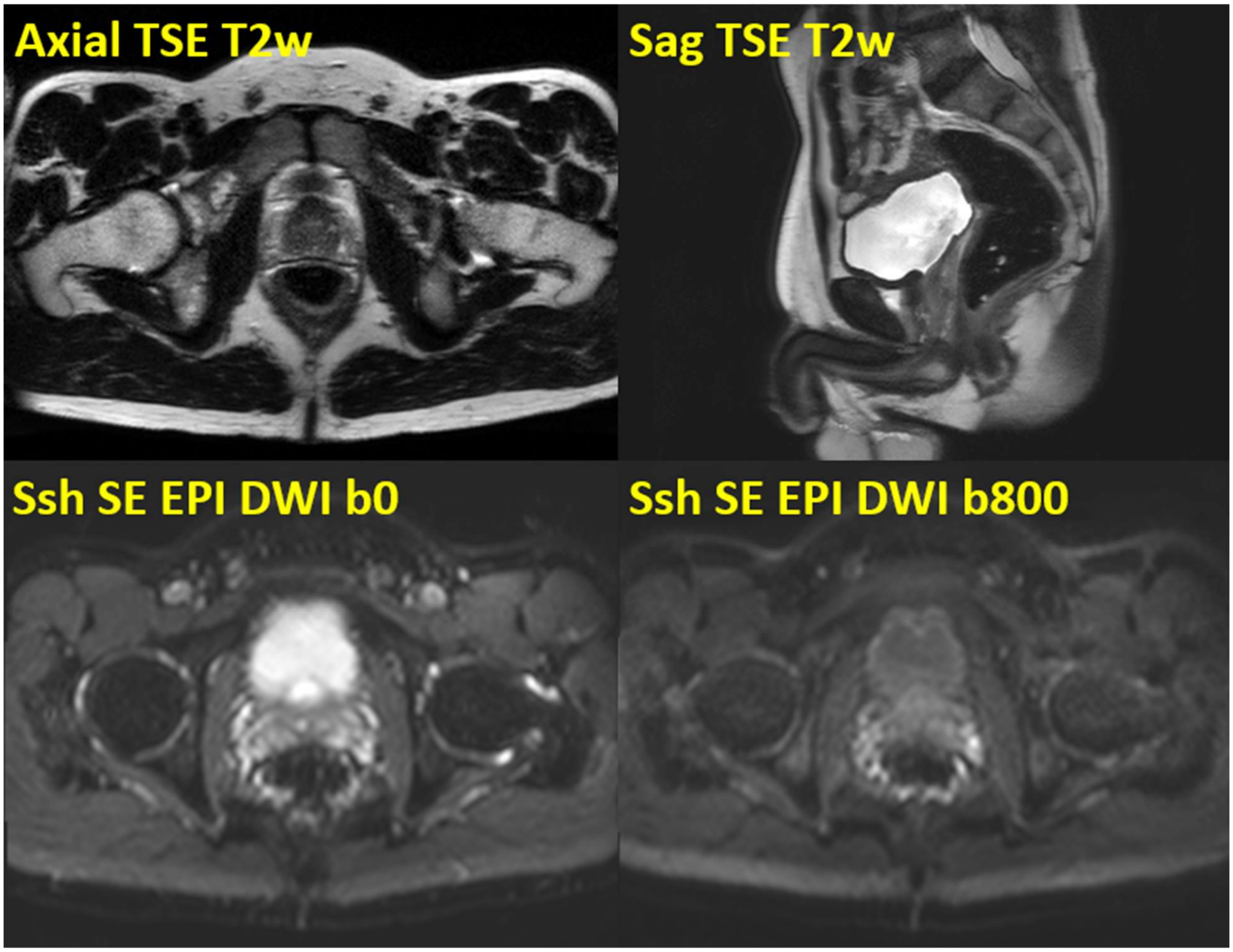

No. 2

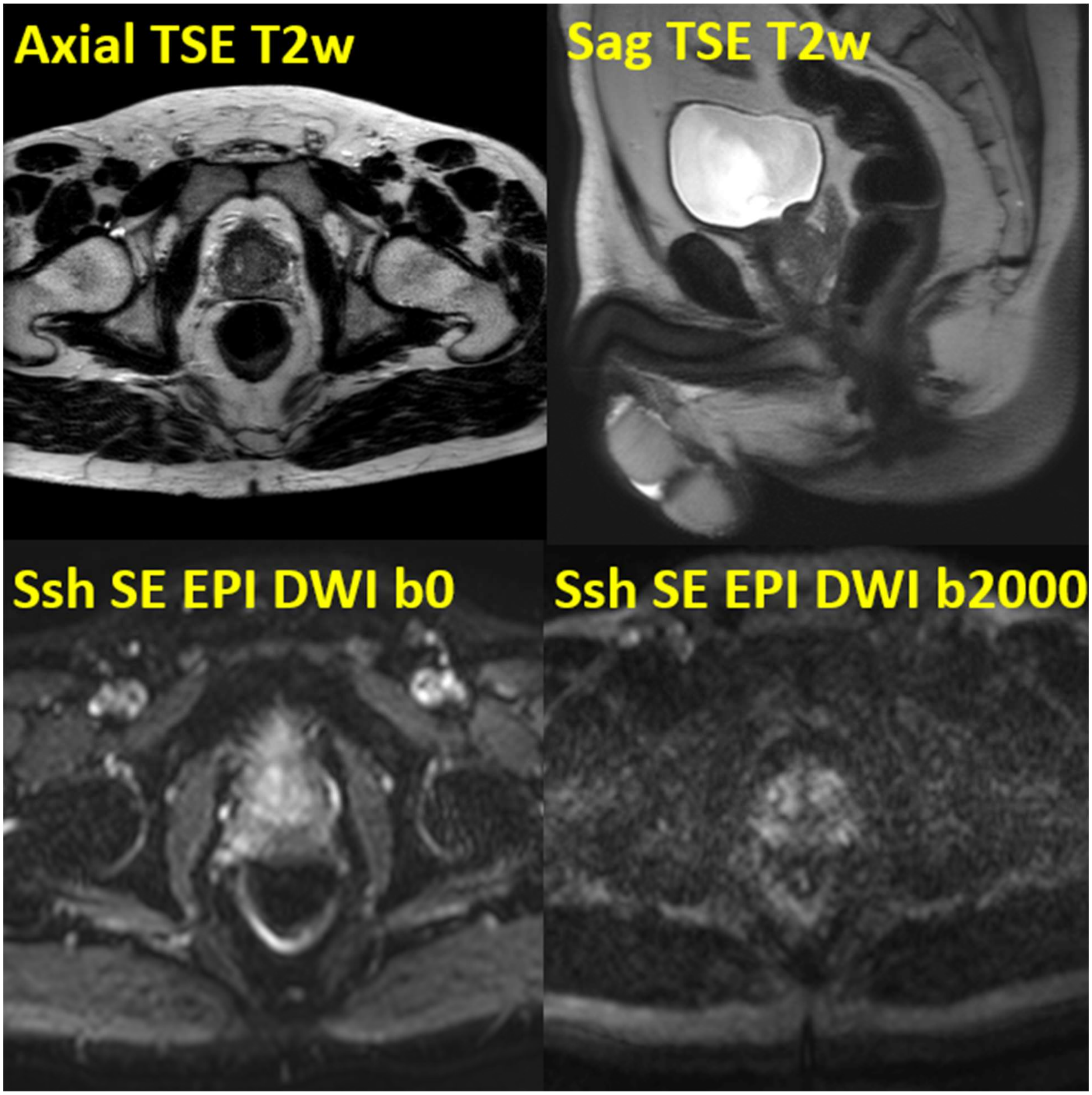

No. 3

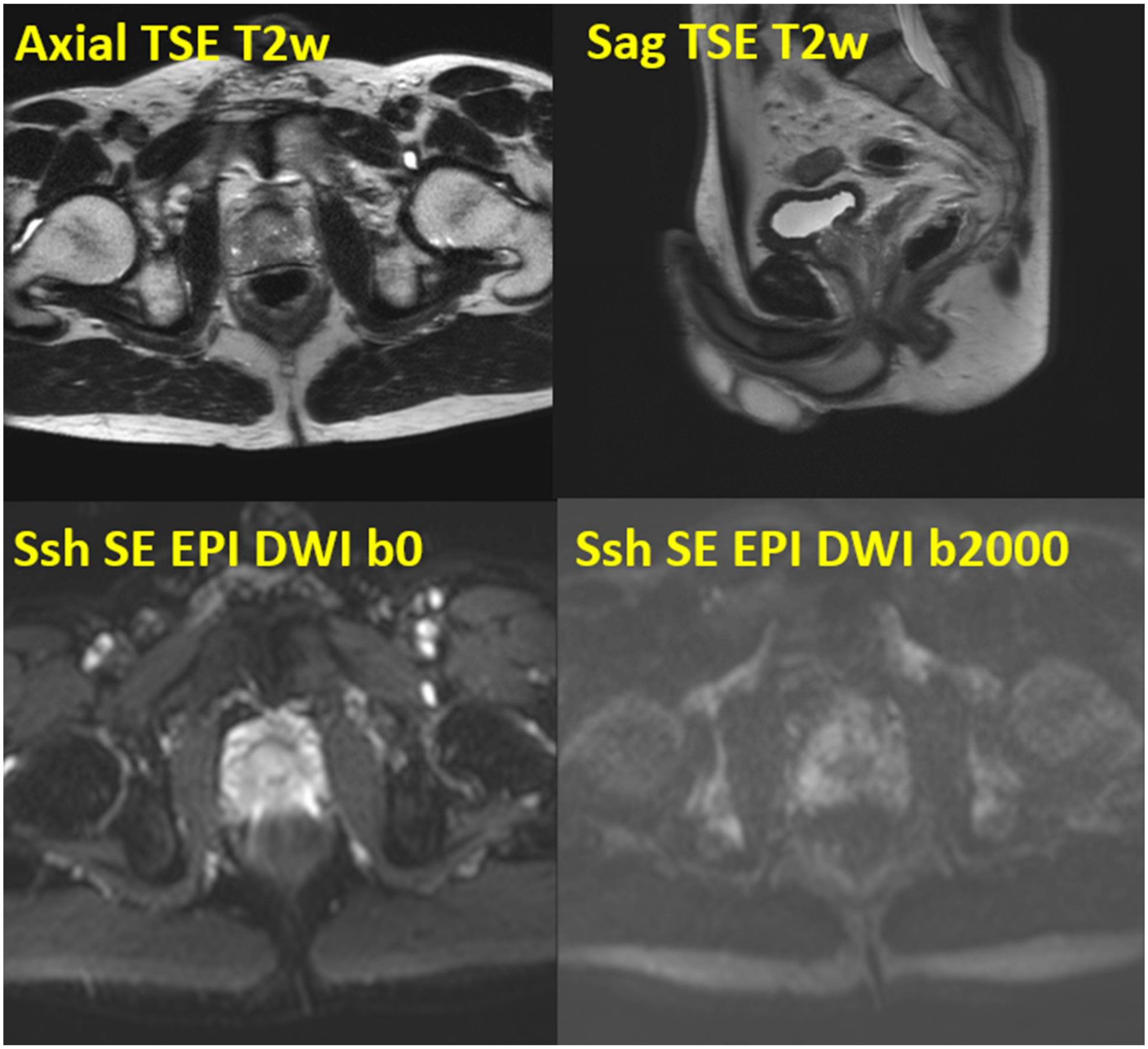

No. 4

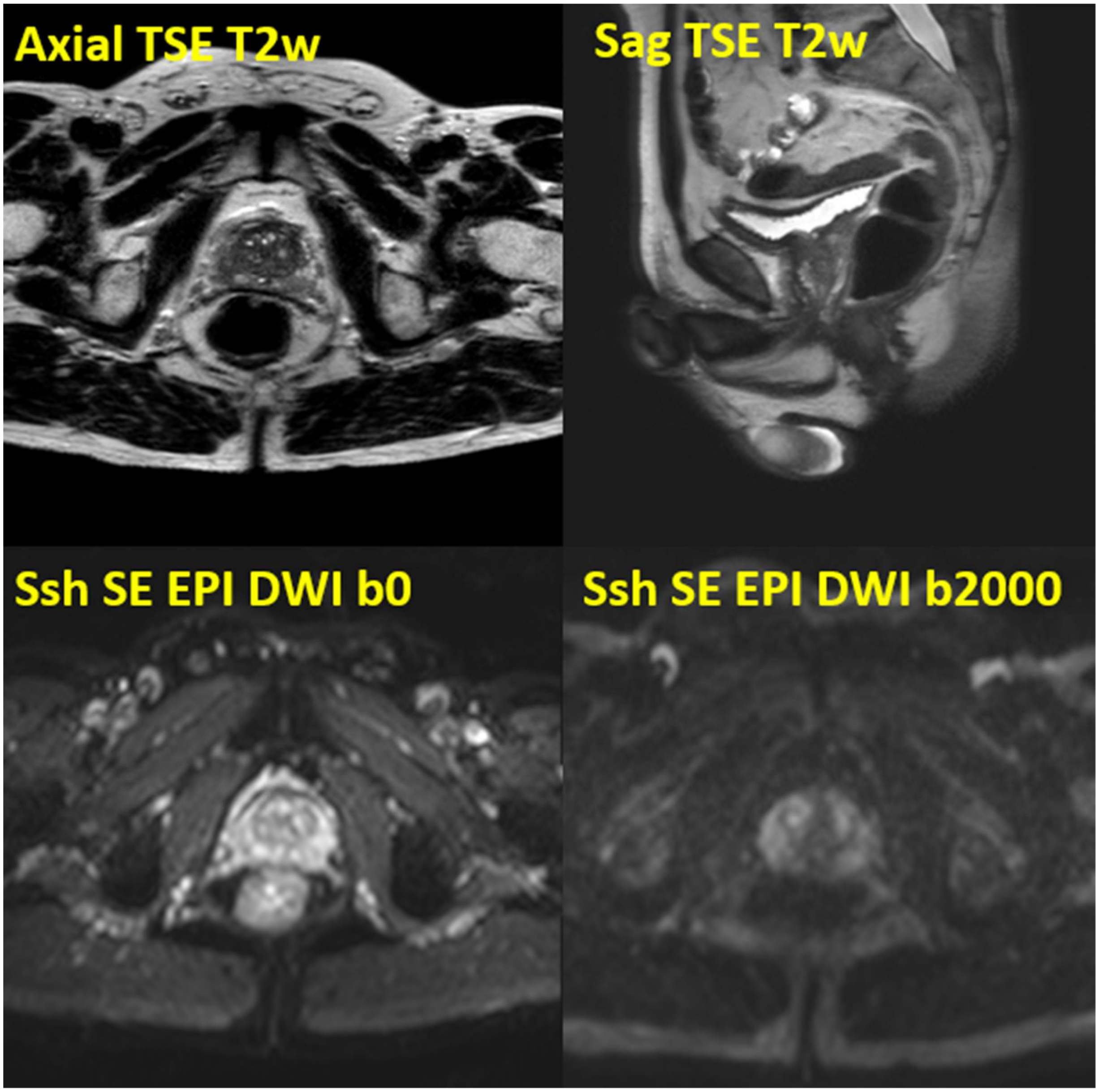

No. 5

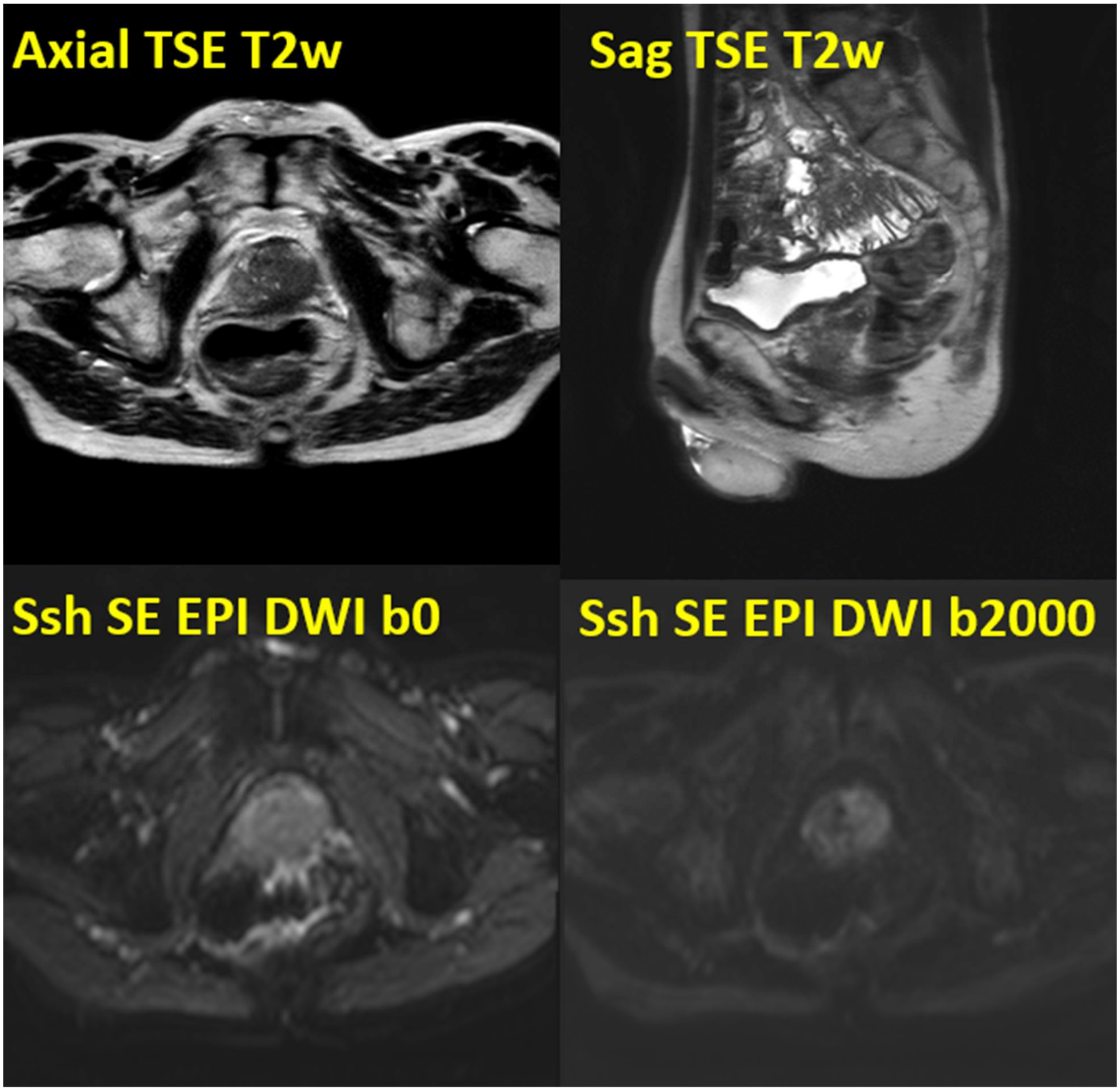

Supplement: Supplementary file 1 — Supplementary information. [file 41598_2020_66255_MOESM1_ESM.pdf]
