# Supplementary material for: Prediction of prostate cancer aggressiveness using 18F-Fluciclovine (FACBC) PET and multisequence multiparametric MRI
Source: Sci Rep. 2020 Jun 10;10:9407. doi: 10.1038/s41598-020-66255-8 (PMC7287051; doi:10.1038/s41598-020-66255-8)
Supplement: Supplementary file 2 — Supplementary information2. [file 41598_2020_66255_MOESM2_ESM.pdf]

Table of contents

\\Vanha Verio
RESEARCH
Prostata
FLUCIPRO
localizer_sag_tse
localizer_tra_tse
localizer_sag_tse=ref
localizer_cor_tse=ref
t2_tse_sag_320_p2
t2_tse_tra_320_p2
t2_tse_cor_320_p2
diff_tra_b0_100_200_350_500
b_1500
b_2000
csi3d_P_12x12x12_8x8x8_I_NA4
d_16b_equally
t1_vibe_tra_FA2
t1_vibe_tra_FA5
t1_vibe_tra_FA8
t1_vibe_tra_FA10
t1_vibe_tra_FA15
t1_vibe_tra_FA15_DYN

\\Vanha Verio\RESEARCH\Prostata\FLUCIPRO\localizer_sag_tse
TA: 0:21 PM: ISO Voxel size: 1.4×1.4×4.0 mmPAT: 2 Rel. SNR: 1.00 : tse

Properties

Prio recon	Off
Load images to viewer	On
Inline movie	Off
Auto store images	On
Load images to stamp segments	Off
Load images to graphic segments	Off
Auto open inline display	Off
Auto close inline display	Off
Start measurement without further preparation	Off
Wait for user to start	Off
Start measurements	Single measurement

Routine

Slice group	1
Slices	14
Dist. factor	50 %
Position	Isocenter
Orientation	Sagittal
Phase enc. dir.	A >> P
AutoAlign	---
Phase oversampling	0 %
FoV read	360 mm
FoV phase	100.0 %
Slice thickness	4.0 mm
TR	3990.0 ms
TE	85 ms
Averages	1
Concatenations	1
Filter	Distortion Corr.(2D)
Coil elements	BO1-3;SP4-7

Contrast - Common

TR	3990.0 ms
TE	85 ms
MTC	Off
Magn. preparation	None
Flip angle	150 deg
Fat suppr.	None
Water suppr.	None
Restore magn.	Off

Contrast - Dynamic

Averages	1
Averaging mode	Long term
Reconstruction	Magnitude
Measurements	1
Multiple series	Each measurement

Resolution - Common

FoV read	360 mm
FoV phase	100.0 %
Slice thickness	4.0 mm
Base resolution	256
Phase resolution	70 %
Phase partial Fourier	Off
Trajectory	Cartesian
Interpolation	Off

Resolution - iPAT

PAT mode	GRAPPA
Accel. factor PE	2
Ref. lines PE	27
Reference scan mode	Integrated

Resolution - Filter Image

Image Filter	Off
Distortion Corr.	On
Mode	2D
Unfiltered images	Off
Prescan Normalize	Off
Normalize	Off
B1 filter	Off

Resolution - Filter Rawdata

Raw filter	Off
Elliptical filter	Off

Geometry - Common

Slice group	1
Slices	14
Dist. factor	50 %
Position	Isocenter
Orientation	Sagittal
Phase enc. dir.	A >> P
FoV read	360 mm
FoV phase	100.0 %
Slice thickness	4.0 mm
TR	3990.0 ms
Multi-slice mode	Interleaved
Series	Interleaved
Concatenations	1

Geometry - AutoAlign

Slice group	1
Position	Isocenter
Orientation	Sagittal
Phase enc. dir.	A >> P
AutoAlign	---
Initial Position	Isocenter
L	0.0 mm
P	0.0 mm
H	0.0 mm
Initial Rotation	0.00 deg
Initial Orientation	Transversal

Geometry - Saturation

Fat suppr.	None
Water suppr.	None
Restore magn.	Off
Special sat.	None

Geometry - Navigator

Geometry - Tim Planning Suite

Set-n-Go Protocol	Off
Table position	H
Table position	0 mm
Inline Composing	Off

System - Miscellaneous

Positioning mode	ISO
Table position	H
Table position	0 mm
MSMA	S - C - T
Sagittal	R >> L
Coronal	A >> P
Transversal	F >> H
Coil Combine Mode	Adaptive Combine
Save uncombined	Off
Matrix Optimization	Off
Coil Focus	Flat
AutoAlign	---
Coil Select Mode	Off - AutoCoilSelect

System - Adjustments

B0 Shim mode	Tune up
B1 Shim mode	TrueForm
Adjust with body coil	Off
Confirm freq. adjustment	Off
Assume Dominant Fat	Off
Assume Silicone	Off
Adjustment Tolerance	Auto

System - Adjust Volume

Position	Isocenter
Orientation	Transversal
Rotation	0.00 deg
A >> P	263 mm
R >> L	350 mm
F >> H	350 mm
Reset	Off

System - Tx/Rx

Frequency 1H	123.219268 MHz
Correction factor	1
Gain	High
Img. Scale Cor.	1.000
Reset	Off
? Ref. amplitude 1H	0.000 V

Physio - Signal1

1st Signal/Mode	None
TR	3990.0 ms
Concatenations	1

Physio - Cardiac

Magn. preparation	None
Fat suppr.	None
Dark blood	Off
FoV read	360 mm
FoV phase	100.0 %
Phase resolution	70 %
Trajectory	Cartesian

Physio - PACE

Resp. control	Off
Concatenations	1

Inline - Common

Subtract	Off
Measurements	1
StdDev	Off
Save original images	On

Inline - MIP

MIP-Sag	Off
MIP-Cor	Off
MIP-Tra	Off
MIP-Time	Off
Save original images	On

Inline - Composing

Inline Composing	Off
Distortion Corr.	On
Mode	2D
Unfiltered images	Off

Sequence - Part 1

Introduction	On
Dimension	2D
Compensate T2 decay	Off
Reduce Motion Sens.	On
Contrasts	1
Flow comp.	No
Multi-slice mode	Interleaved
Free echo spacing	Off
Echo spacing	10.7 ms
Bandwidth	199 Hz/Px

Sequence - Part 2

Define	Turbo factor
Echo trains per slice	4
Phase correction	Automatic
Acoustic noise reduction	None
RF pulse type	Low SAR
Gradient mode	Normal
Hyperecho	Off
WARP	Off
Red. EC sensitivity	Off
Turbo factor	26

Sequence - Assistant

Mode	Off
Allowed delay	30 s

\\Vanha Verio\RESEARCH\Prostata\FLUCIPRO\localizer_tra_tse
TA: 0:21 PM: ISO Voxel size: 1.3×1.3×5.0 mmPAT: 2 Rel. SNR: 1.00 : tse

Properties

Prio recon	Off
Load images to viewer	On
Inline movie	Off
Auto store images	On
Load images to stamp segments	Off
Load images to graphic segments	Off
Auto open inline display	Off
Auto close inline display	Off
Start measurement without further preparation	Off
Wait for user to start	Off
Start measurements	Single measurement

Routine

Slice group	1
Slices	12
Dist. factor	60 %
Position	R11.6 P33.5 F0.5 mm
Orientation	Transversal
Phase enc. dir.	A >> P
AutoAlign	---
Phase oversampling	50 %
FoV read	250 mm
FoV phase	100.0 %
Slice thickness	5.0 mm
TR	3290.0 ms
TE	96 ms
Averages	1
Concatenations	1
Filter	Distortion Corr.(2D), Elliptical filter
Coil elements	BO1-3;SP6

Contrast - Common

TR	3290.0 ms
TE	96 ms
MTC	Off
Magn. preparation	None
Flip angle	150 deg
Fat suppr.	None
Water suppr.	None
Restore magn.	Off

Contrast - Dynamic

Averages	1
Averaging mode	Long term
Reconstruction	Magnitude
Measurements	1
Multiple series	Each measurement

Resolution - Common

FoV read	250 mm
FoV phase	100.0 %
Slice thickness	5.0 mm
Base resolution	192
Phase resolution	75 %
Phase partial Fourier	Off
Trajectory	Cartesian
Interpolation	Off

Resolution - iPAT

PAT mode	GRAPPA
Accel. factor PE	2
Ref. lines PE	34
Reference scan mode	Integrated

Resolution - Filter Image

Image Filter	Off
Distortion Corr.	On
Mode	2D
Unfiltered images	Off
Prescan Normalize	Off
Normalize	Off
B1 filter	Off

Resolution - Filter Rawdata

Raw filter	Off
Elliptical filter	On

Geometry - Common

Slice group	1
Slices	12
Dist. factor	60 %
Position	R11.6 P33.5 F0.5 mm
Orientation	Transversal
Phase enc. dir.	A >> P
FoV read	250 mm
FoV phase	100.0 %
Slice thickness	5.0 mm
TR	3290.0 ms
Multi-slice mode	Interleaved
Series	Interleaved
Concatenations	1

Geometry - AutoAlign

Slice group	1
Position	R11.6 P33.5 F0.5 mm
Orientation	Transversal
Phase enc. dir.	A >> P
AutoAlign	---
Initial Position	Isocenter
L	0.0 mm
P	0.0 mm
H	0.0 mm
Initial Rotation	0.00 deg
Initial Orientation	Transversal

Geometry - Saturation

Fat suppr.	None
Water suppr.	None
Restore magn.	Off
Special sat.	None

Geometry - Navigator

Geometry - Tim Planning Suite

Set-n-Go Protocol	Off
Table position	H
Table position	0 mm
Inline Composing	Off

System - Miscellaneous

Positioning mode	ISO
Table position	H
Table position	0 mm
MSMA	S - C - T
Sagittal	R >> L
Coronal	A >> P
Transversal	F >> H
Coil Combine Mode	Adaptive Combine
Save uncombined	Off
Matrix Optimization	Off
Coil Focus	Flat
AutoAlign	---
Coil Select Mode	Default

System - Adjustments

B0 Shim mode	Tune up
B1 Shim mode	TrueForm
Adjust with body coil	Off
Confirm freq. adjustment	Off
Assume Dominant Fat	Off
Assume Silicone	Off
Adjustment Tolerance	Auto

System - Adjust Volume

Position	Isocenter
Orientation	Transversal
Rotation	0.00 deg
A >> P	263 mm
R >> L	350 mm
F >> H	350 mm
Reset	Off

System - Tx/Rx

Frequency 1H	123.219268 MHz
Correction factor	1
Gain	High
Img. Scale Cor.	1.000
Reset	Off
? Ref. amplitude 1H	0.000 V

Physio - Signal1

1st Signal/Mode	None
TR	3290.0 ms
Concatenations	1

Physio - Cardiac

Magn. preparation	None
Fat suppr.	None
Dark blood	Off
FoV read	250 mm
FoV phase	100.0 %
Phase resolution	75 %
Trajectory	Cartesian

Physio - PACE

Resp. control	Off
Concatenations	1

Inline - Common

Subtract	Off
Measurements	1
StdDev	Off
Save original images	On

Inline - MIP

MIP-Sag	Off
MIP-Cor	Off
MIP-Tra	Off
MIP-Time	Off
Save original images	On

Inline - Composing

Inline Composing	Off
Distortion Corr.	On
Mode	2D
Unfiltered images	Off

Sequence - Part 1

Introduction	On
Dimension	2D
Compensate T2 decay	Off
Reduce Motion Sens.	On
Contrasts	1
Flow comp.	No
Multi-slice mode	Interleaved
Free echo spacing	Off
Echo spacing	10.6 ms
Bandwidth	200 Hz/Px

Sequence - Part 2

Define	Turbo factor
Echo trains per slice	5
Phase correction	Automatic
Acoustic noise reduction	None
RF pulse type	Low SAR
Gradient mode	Normal
Hyperecho	Off
WARP	Off
Red. EC sensitivity	Off
Turbo factor	25

Sequence - Assistant

Mode	Off
Allowed delay	30 s

\\Vanha Verio\RESEARCH\Prostata\FLUCIPRO\localizer_sag_tse=ref
TA: 0:21 PM: FIX Voxel size: 1.4×1.4×4.0 mmPAT: 2 Rel. SNR: 1.00 : tse

Properties

Prio recon	Off
Load images to viewer	On
Inline movie	Off
Auto store images	On
Load images to stamp segments	Off
Load images to graphic segments	Off
Auto open inline display	Off
Auto close inline display	Off
Start measurement without further preparation	Off
Wait for user to start	Off
Start measurements	Single measurement

Routine

Slice group	1
Slices	14
Dist. factor	50 %
Position	Isocenter
Orientation	Sagittal
Phase enc. dir.	A >> P
AutoAlign	---
Phase oversampling	0 %
FoV read	360 mm
FoV phase	100.0 %
Slice thickness	4.0 mm
TR	3990.0 ms
TE	85 ms
Averages	1
Concatenations	1
Filter	None
Coil elements	BO1-3;SP5-7

Contrast - Common

TR	3990.0 ms
TE	85 ms
MTC	Off
Magn. preparation	None
Flip angle	150 deg
Fat suppr.	None
Water suppr.	None
Restore magn.	Off

Contrast - Dynamic

Averages	1
Averaging mode	Long term
Reconstruction	Magnitude
Measurements	1
Multiple series	Each measurement

Resolution - Common

FoV read	360 mm
FoV phase	100.0 %
Slice thickness	4.0 mm
Base resolution	256
Phase resolution	70 %
Phase partial Fourier	Off
Trajectory	Cartesian
Interpolation	Off

Resolution - iPAT

PAT mode	GRAPPA
Accel. factor PE	2
Ref. lines PE	27
Reference scan mode	Integrated

Resolution - Filter Image

Image Filter	Off
Distortion Corr.	Off
Prescan Normalize	Off
Normalize	Off
B1 filter	Off

Resolution - Filter Rawdata

Raw filter	Off
Elliptical filter	Off

Geometry - Common

Slice group	1
Slices	14
Dist. factor	50 %
Position	Isocenter
Orientation	Sagittal
Phase enc. dir.	A >> P
FoV read	360 mm
FoV phase	100.0 %
Slice thickness	4.0 mm
TR	3990.0 ms
Multi-slice mode	Interleaved
Series	Interleaved
Concatenations	1

Geometry - AutoAlign

Slice group	1
Position	Isocenter
Orientation	Sagittal
Phase enc. dir.	A >> P
AutoAlign	---
Initial Position	Isocenter
L	0.0 mm
P	0.0 mm
H	0.0 mm
Initial Rotation	0.00 deg
Initial Orientation	Transversal

Geometry - Saturation

Fat suppr.	None
Water suppr.	None
Restore magn.	Off
Special sat.	None

Geometry - Navigator

Geometry - Tim Planning Suite

Set-n-Go Protocol	Off
Table position	H
Table position	0 mm
Inline Composing	Off

System - Miscellaneous

Positioning mode	FIX
Table position	H
Table position	0 mm
MSMA	S - C - T
Sagittal	R >> L
Coronal	A >> P
Transversal	F >> H
Coil Combine Mode	Adaptive Combine
Save uncombined	Off
Matrix Optimization	Off
Coil Focus	Flat
AutoAlign	---
Coil Select Mode	Default

System - Adjustments

B0 Shim mode	Tune up
B1 Shim mode	TrueForm
Adjust with body coil	Off
Confirm freq. adjustment	Off
Assume Dominant Fat	Off
Assume Silicone	Off
Adjustment Tolerance	Auto

System - Adjust Volume

Position	Isocenter
Orientation	Transversal
Rotation	0.00 deg
A >> P	263 mm
R >> L	350 mm
F >> H	350 mm
Reset	Off

System - Tx/Rx

Frequency 1H	123.219268 MHz
Correction factor	1
Gain	High
Img. Scale Cor.	1.000
Reset	Off
? Ref. amplitude 1H	0.000 V

Physio - Signal1

1st Signal/Mode	None
TR	3990.0 ms
Concatenations	1

Physio - Cardiac

Magn. preparation	None
Fat suppr.	None
Dark blood	Off
FoV read	360 mm
FoV phase	100.0 %
Phase resolution	70 %
Trajectory	Cartesian

Physio - PACE

Resp. control	Off
Concatenations	1

Inline - Common

Subtract	Off
Measurements	1
StdDev	Off
Save original images	On

Inline - MIP

MIP-Sag	Off
MIP-Cor	Off
MIP-Tra	Off
MIP-Time	Off
Save original images	On

Inline - Composing

Inline Composing	Off
Distortion Corr.	Off

Sequence - Part 1

Introduction	On
Dimension	2D
Compensate T2 decay	Off
Reduce Motion Sens.	On
Contrasts	1
Flow comp.	No
Multi-slice mode	Interleaved
Free echo spacing	Off
Echo spacing	10.7 ms
Bandwidth	199 Hz/Px

Sequence - Part 2

Define	Turbo factor
Echo trains per slice	4
Phase correction	Automatic
Acoustic noise reduction	None
RF pulse type	Low SAR
Gradient mode	Normal
Hyperecho	Off
WARP	Off
Red. EC sensitivity	Off
Turbo factor	26

Sequence - Assistant

Mode	Off
Allowed delay	30 s

\\Vanha Verio\RESEARCH\Prostata\FLUCIPRO\localizer_cor_tse=ref
TA: 0:20 PM: FIX Voxel size: 1.0×1.0×3.0 mmPAT: 2 Rel. SNR: 1.00 : tse

Properties

Prio recon	Off
Load images to viewer	On
Inline movie	Off
Auto store images	On
Load images to stamp segments	Off
Load images to graphic segments	Off
Auto open inline display	Off
Auto close inline display	Off
Start measurement without further preparation	Off
Wait for user to start	Off
Start measurements	Single measurement

Routine

Slice group	1
Slices	10
Dist. factor	0 %
Position	L0.0 P25.2 H8.7 mm
Orientation	Coronal
Phase enc. dir.	R >> L
AutoAlign	---
Phase oversampling	100 %
FoV read	200 mm
FoV phase	100.0 %
Slice thickness	3.0 mm
TR	3000.0 ms
TE	85 ms
Averages	1
Concatenations	1
Filter	Prescan Normalize
Coil elements	BO1-3;SP5,7

Contrast - Common

TR	3000.0 ms
TE	85 ms
MTC	Off
Magn. preparation	None
Flip angle	150 deg
Fat suppr.	None
Water suppr.	None
Restore magn.	Off

Contrast - Dynamic

Averages	1
Averaging mode	Long term
Reconstruction	Magnitude
Measurements	1
Multiple series	Each measurement

Resolution - Common

FoV read	200 mm
FoV phase	100.0 %
Slice thickness	3.0 mm
Base resolution	192
Phase resolution	58 %
Phase partial Fourier	Off
Trajectory	Cartesian
Interpolation	Off

Resolution - iPAT

PAT mode	GRAPPA
Accel. factor PE	2
Ref. lines PE	27
Reference scan mode	Integrated

Resolution - Filter Image

Image Filter	Off
Distortion Corr.	Off
Prescan Normalize	On
Unfiltered images	Off
Normalize	Off
B1 filter	Off

Resolution - Filter Rawdata

Raw filter	Off
Elliptical filter	Off

Geometry - Common

Slice group	1
Slices	10
Dist. factor	0 %
Position	L0.0 P25.2 H8.7 mm
Orientation	Coronal
Phase enc. dir.	R >> L
FoV read	200 mm
FoV phase	100.0 %
Slice thickness	3.0 mm
TR	3000.0 ms
Multi-slice mode	Interleaved
Series	Interleaved
Concatenations	1

Geometry - AutoAlign

Slice group	1
Position	L0.0 P25.2 H8.7 mm
Orientation	Coronal
Phase enc. dir.	R >> L
AutoAlign	---
Initial Position	Isocenter
L	0.0 mm
P	0.0 mm
H	0.0 mm
Initial Rotation	0.00 deg
Initial Orientation	Transversal

Geometry - Saturation

Fat suppr.	None
Water suppr.	None
Restore magn.	Off
Special sat.	None

Geometry - Navigator

Geometry - Tim Planning Suite

Set-n-Go Protocol	Off
Table position	H
Table position	0 mm
Inline Composing	Off

System - Miscellaneous

Positioning mode	FIX
Table position	H
Table position	0 mm
MSMA	S - C - T
Sagittal	R >> L
Coronal	A >> P
Transversal	F >> H
Coil Combine Mode	Adaptive Combine
Save uncombined	Off
Matrix Optimization	Off
Coil Focus	Flat
AutoAlign	---
Coil Select Mode	Default

System - Adjustments

B0 Shim mode	Tune up
B1 Shim mode	TrueForm
Adjust with body coil	Off
Confirm freq. adjustment	Off
Assume Dominant Fat	Off
Assume Silicone	Off
Adjustment Tolerance	Auto

System - Adjust Volume

Position	Isocenter
Orientation	Transversal
Rotation	0.00 deg
A >> P	263 mm
R >> L	350 mm
F >> H	350 mm
Reset	Off

System - Tx/Rx

Frequency 1H	123.219268 MHz
Correction factor	1
Gain	High
Img. Scale Cor.	1.000
Reset	Off
? Ref. amplitude 1H	0.000 V

Physio - Signal1

1st Signal/Mode	None
TR	3000.0 ms
Concatenations	1

Physio - Cardiac

Magn. preparation	None
Fat suppr.	None
Dark blood	Off
FoV read	200 mm
FoV phase	100.0 %
Phase resolution	58 %
Trajectory	Cartesian

Physio - PACE

Resp. control	Off
Concatenations	1

Inline - Common

Subtract	Off
Measurements	1
StdDev	Off
Save original images	On

Inline - MIP

MIP-Sag	Off
MIP-Cor	Off
MIP-Tra	Off
MIP-Time	Off
Save original images	On

Inline - Composing

Inline Composing	Off
Distortion Corr.	Off

Sequence - Part 1

Introduction	On
Dimension	2D
Compensate T2 decay	Off
Reduce Motion Sens.	On
Contrasts	1
Flow comp.	No
Multi-slice mode	Interleaved
Free echo spacing	Off
Echo spacing	10.7 ms
Bandwidth	199 Hz/Px

Sequence - Part 2

Define	Turbo factor
Echo trains per slice	5
Phase correction	Automatic
Acoustic noise reduction	None
RF pulse type	Low SAR
Gradient mode	Normal
Hyperecho	Off
WARP	Off
Red. EC sensitivity	Off
Turbo factor	25

Sequence - Assistant

Mode	Off
Allowed delay	30 s

\\Vanha Verio\RESEARCH\Prostata\FLUCIPRO\t2_tse_sag_320_p2

TA: 2:28 PM: REF Voxel size: 0.6×0.6×3.0 mmPAT: 2 Rel. SNR: 1.00 : tse

Properties

Prio recon	Off
Load images to viewer	On
Inline movie	Off
Auto store images	On
Load images to stamp segments	Off
Load images to graphic segments	Off
Auto open inline display	Off
Auto close inline display	Off
Start measurement without further preparation	Off
Wait for user to start	Off
Start measurements	Single measurement

Routine

Slice group	1
Slices	30
Dist. factor	0 %
Position	L4.8 P36.8 H4.8 mm
Orientation	Sagittal
Phase enc. dir.	A >> P
AutoAlign	---
Phase oversampling	43 %
FoV read	200 mm
FoV phase	100.0 %
Slice thickness	3.0 mm
TR	8640.0 ms
TE	101 ms
Averages	2
Concatenations	1
Filter	Prescan Normalize
Coil elements	BO1-3;SP5-7

Contrast - Common

TR	8640.0 ms
TE	101 ms
MTC	Off
Magn. preparation	None
Flip angle	150 deg
Fat suppr.	None
Water suppr.	None
Restore magn.	Off

Contrast - Dynamic

Averages	2
Averaging mode	Long term
Reconstruction	Magnitude
Measurements	1
Multiple series	Each measurement

Resolution - Common

FoV read	200 mm
FoV phase	100.0 %
Slice thickness	3.0 mm
Base resolution	320
Phase resolution	85 %
Phase partial Fourier	Off
Trajectory	Cartesian
Interpolation	Off

Resolution - iPAT

PAT mode	GRAPPA
Accel. factor PE	2
Ref. lines PE	32
Reference scan mode	Self-calibration

Resolution - Filter Image

Image Filter	Off
Distortion Corr.	Off
Prescan Normalize	On
Unfiltered images	Off
Normalize	Off
B1 filter	Off

Resolution - Filter Rawdata

Raw filter	Off
Elliptical filter	Off

Geometry - Common

Slice group	1
Slices	30
Dist. factor	0 %
Position	L4.8 P36.8 H4.8 mm
Orientation	Sagittal
Phase enc. dir.	A >> P
FoV read	200 mm
FoV phase	100.0 %
Slice thickness	3.0 mm
TR	8640.0 ms
Multi-slice mode	Interleaved
Series	Interleaved
Concatenations	1

Geometry - AutoAlign

Slice group	1
Position	L4.8 P36.8 H4.8 mm
Orientation	Sagittal
Phase enc. dir.	A >> P
AutoAlign	---
Initial Position	Isocenter
L	0.0 mm
P	0.0 mm
H	0.0 mm
Initial Rotation	0.00 deg
Initial Orientation	Transversal

Geometry - Saturation

Fat suppr.	None
Water suppr.	None
Restore magn.	Off
Special sat.	None

Geometry - Navigator

Geometry - Tim Planning Suite

Set-n-Go Protocol	Off
Table position	H
Table position	0 mm
Inline Composing	Off

System - Miscellaneous

Positioning mode	REF
Table position	H
Table position	0 mm
MSMA	S - C - T
Sagittal	R >> L
Coronal	A >> P
Transversal	F >> H
Coil Combine Mode	Adaptive Combine
Save uncombined	Off
Matrix Optimization	Off
Coil Focus	Flat
AutoAlign	---
Coil Select Mode	Default

System - Adjustments

B0 Shim mode	Tune up
B1 Shim mode	TrueForm
Adjust with body coil	Off
Confirm freq. adjustment	Off
Assume Dominant Fat	Off
Assume Silicone	Off
Adjustment Tolerance	Auto

System - Adjust Volume

Position	Isocenter
Orientation	Transversal
Rotation	0.00 deg
A >> P	263 mm
R >> L	350 mm
F >> H	350 mm
Reset	Off

System - Tx/Rx

Frequency 1H	123.219268 MHz
Correction factor	1
Gain	High
Img. Scale Cor.	1.000
Reset	Off
? Ref. amplitude 1H	0.000 V

Physio - Signal1

1st Signal/Mode	None
TR	8640.0 ms
Concatenations	1

Physio - Cardiac

Magn. preparation	None
Fat suppr.	None
Dark blood	Off
FoV read	200 mm
FoV phase	100.0 %
Phase resolution	85 %
Trajectory	Cartesian

Physio - PACE

Resp. control	Off
Concatenations	1

Inline - Common

Subtract	Off
Measurements	1
StdDev	Off
Save original images	On

Inline - MIP

MIP-Sag	Off
MIP-Cor	Off
MIP-Tra	Off
MIP-Time	Off
Save original images	On

Inline - Composing

Inline Composing	Off
Distortion Corr.	Off

Sequence - Part 1

Introduction	On
Dimension	2D
Compensate T2 decay	Off
Reduce Motion Sens.	On
Contrasts	1
Flow comp.	No
Multi-slice mode	Interleaved
Free echo spacing	Off
Echo spacing	11.2 ms
Bandwidth	200 Hz/Px

Sequence - Part 2

Define	Turbo factor
Echo trains per slice	8
Phase correction	Automatic
Acoustic noise reduction	None
RF pulse type	Low SAR
Gradient mode	Normal
Hyperecho	Off
WARP	Off
Red. EC sensitivity	Off
Turbo factor	25

Sequence - Assistant

Mode	Off
Allowed delay	30 s

\\Vanha Verio\RESEARCH\Prostata\FLUCIPRO\t2_tse_tra_320_p2

TA: 2:16 PM: REF Voxel size: 0.6×0.6×3.0 mmPAT: 2 Rel. SNR: 1.00 : tse

Properties

Prio recon	Off
Load images to viewer	On
Inline movie	Off
Auto store images	On
Load images to stamp segments	Off
Load images to graphic segments	Off
Auto open inline display	Off
Auto close inline display	Off
Start measurement without further preparation	Off
Wait for user to start	Off
Start measurements	Single measurement

Routine

Slice group	1
Slices	22
Dist. factor	0 %
Position	R11.6 P33.5 F52.5 mm
Orientation	Transversal
Phase enc. dir.	A >> P
AutoAlign	---
Phase oversampling	50 %
FoV read	200 mm
FoV phase	100.0 %
Slice thickness	3.0 mm
TR	6400.0 ms
TE	101 ms
Averages	2
Concatenations	1
Filter	Prescan Normalize
Coil elements	BO1-3;SP6

Contrast - Common

TR	6400.0 ms
TE	101 ms
MTC	Off
Magn. preparation	None
Flip angle	150 deg
Fat suppr.	None
Water suppr.	None
Restore magn.	Off

Contrast - Dynamic

Averages	2
Averaging mode	Long term
Reconstruction	Magnitude
Measurements	1
Multiple series	Each measurement

Resolution - Common

FoV read	200 mm
FoV phase	100.0 %
Slice thickness	3.0 mm
Base resolution	320
Phase resolution	97 %
Phase partial Fourier	Off
Trajectory	Cartesian
Interpolation	Off

Resolution - iPAT

PAT mode	GRAPPA
Accel. factor PE	2
Ref. lines PE	32
Reference scan mode	Self-calibration

Resolution - Filter Image

Image Filter	Off
Distortion Corr.	Off
Prescan Normalize	On
Unfiltered images	Off
Normalize	Off
B1 filter	Off

Resolution - Filter Rawdata

Raw filter	Off
Elliptical filter	Off

Geometry - Common

Slice group	1
Slices	22
Dist. factor	0 %
Position	R11.6 P33.5 F52.5 mm
Orientation	Transversal
Phase enc. dir.	A >> P
FoV read	200 mm
FoV phase	100.0 %
Slice thickness	3.0 mm
TR	6400.0 ms
Multi-slice mode	Interleaved
Series	Interleaved
Concatenations	1

Geometry - AutoAlign

Slice group	1
Position	R11.6 P33.5 F52.5 mm
Orientation	Transversal
Phase enc. dir.	A >> P
AutoAlign	---
Initial Position	Isocenter
L	0.0 mm
P	0.0 mm
F	0.0 mm
Initial Rotation	0.00 deg
Initial Orientation	Transversal

Geometry - Saturation

Fat suppr.	None
Water suppr.	None
Restore magn.	Off
Special sat.	None

Geometry - Navigator

Geometry - Tim Planning Suite

Set-n-Go Protocol	Off
Table position	F
Table position	52 mm
Inline Composing	Off

System - Miscellaneous

Positioning mode	REF
Table position	F
Table position	52 mm
MSMA	S - C - T
Sagittal	R >> L
Coronal	A >> P
Transversal	F >> H
Coil Combine Mode	Adaptive Combine
Save uncombined	Off
Matrix Optimization	Off
Coil Focus	Flat
AutoAlign	---
Coil Select Mode	Default

System - Adjustments

B0 Shim mode	Standard
B1 Shim mode	TrueForm
Adjust with body coil	Off
Confirm freq. adjustment	Off
Assume Dominant Fat	Off
Assume Silicone	Off
Adjustment Tolerance	Auto

System - Adjust Volume

Position	R11.6 P33.5 F52.5 mm
Orientation	Transversal
Rotation	0.00 deg
A >> P	200 mm
R >> L	200 mm
F >> H	66 mm
Reset	Off

System - Tx/Rx

Frequency 1H	123.219268 MHz
Correction factor	1
Gain	High
Img. Scale Cor.	1.000
Reset	Off
? Ref. amplitude 1H	0.000 V

Physio - Signal1

1st Signal/Mode	None
TR	6400.0 ms
Concatenations	1

Physio - Cardiac

Magn. preparation	None
Fat suppr.	None
Dark blood	Off
FoV read	200 mm
FoV phase	100.0 %
Phase resolution	97 %
Trajectory	Cartesian

Physio - PACE

Resp. control	Off
Concatenations	1

Inline - Common

Subtract	Off
Measurements	1
StdDev	Off
Save original images	On

Inline - MIP

MIP-Sag	Off
MIP-Cor	Off
MIP-Tra	Off
MIP-Time	Off
Save original images	On

Inline - Composing

Inline Composing	Off
Distortion Corr.	Off

Sequence - Part 1

Introduction	On
Dimension	2D
Compensate T2 decay	Off
Reduce Motion Sens.	On
Contrasts	1
Flow comp.	No
Multi-slice mode	Interleaved
Free echo spacing	Off
Echo spacing	11.2 ms
Bandwidth	200 Hz/Px

Sequence - Part 2

Define	Turbo factor
Echo trains per slice	10
Phase correction	Automatic
Acoustic noise reduction	None
RF pulse type	Low SAR
Gradient mode	Normal
Hyperecho	Off
WARP	Off
Red. EC sensitivity	Off
Turbo factor	25

Sequence - Assistant

Mode	Off
Allowed delay	30 s

\\Vanha Verio\RESEARCH\Prostata\FLUCIPRO\t2_tse_cor_320_p2
TA: 2:47 PM: REF Voxel size: 0.6×0.6×3.0 mmPAT: 2 Rel. SNR: 1.00 : tse

Properties

Prio recon	Off
Load images to viewer	On
Inline movie	Off
Auto store images	On
Load images to stamp segments	Off
Load images to graphic segments	Off
Auto open inline display	Off
Auto close inline display	Off
Start measurement without further preparation	Off
Wait for user to start	Off
Start measurements	Single measurement

Routine

Slice group	1
Slices	25
Dist. factor	0 %
Position	L0.0 P25.2 H8.7 mm
Orientation	Coronal
Phase enc. dir.	R >> L
AutoAlign	---
Phase oversampling	100 %
FoV read	200 mm
FoV phase	100.0 %
Slice thickness	3.0 mm
TR	7200.0 ms
TE	101 ms
Averages	2
Concatenations	1
Filter	Prescan Normalize
Coil elements	BO1-3;SP5-7

Contrast - Common

TR	7200.0 ms
TE	101 ms
MTC	Off
Magn. preparation	None
Flip angle	150 deg
Fat suppr.	None
Water suppr.	None
Restore magn.	Off

Contrast - Dynamic

Averages	2
Averaging mode	Long term
Reconstruction	Magnitude
Measurements	1
Multiple series	Each measurement

Resolution - Common

FoV read	200 mm
FoV phase	100.0 %
Slice thickness	3.0 mm
Base resolution	320
Phase resolution	85 %
Phase partial Fourier	Off
Trajectory	Cartesian
Interpolation	Off

Resolution - iPAT

PAT mode	GRAPPA
Accel. factor PE	2
Ref. lines PE	32
Reference scan mode	Self-calibration

Resolution - Filter Image

Image Filter	Off
Distortion Corr.	Off
Prescan Normalize	On
Unfiltered images	Off
Normalize	Off
B1 filter	Off

Resolution - Filter Rawdata

Raw filter	Off
Elliptical filter	Off

Geometry - Common

Slice group	1
Slices	25
Dist. factor	0 %
Position	L0.0 P25.2 H8.7 mm
Orientation	Coronal
Phase enc. dir.	R >> L
FoV read	200 mm
FoV phase	100.0 %
Slice thickness	3.0 mm
TR	7200.0 ms
Multi-slice mode	Interleaved
Series	Interleaved
Concatenations	1

Geometry - AutoAlign

Slice group	1
Position	L0.0 P25.2 H8.7 mm
Orientation	Coronal
Phase enc. dir.	R >> L
AutoAlign	---
Initial Position	Isocenter
L	0.0 mm
P	0.0 mm
H	0.0 mm
Initial Rotation	0.00 deg
Initial Orientation	Transversal

Geometry - Saturation

Fat suppr.	None
Water suppr.	None
Restore magn.	Off
Special sat.	None

Geometry - Navigator

Geometry - Tim Planning Suite

Set-n-Go Protocol	Off
Table position	H
Table position	0 mm
Inline Composing	Off

System - Miscellaneous

Positioning mode	REF
Table position	H
Table position	0 mm
MSMA	S - C - T
Sagittal	R >> L
Coronal	A >> P
Transversal	F >> H
Coil Combine Mode	Adaptive Combine
Save uncombined	Off
Matrix Optimization	Off
Coil Focus	Flat
AutoAlign	---
Coil Select Mode	Default

System - Adjustments

B0 Shim mode	Tune up
B1 Shim mode	TrueForm
Adjust with body coil	Off
Confirm freq. adjustment	Off
Assume Dominant Fat	Off
Assume Silicone	Off
Adjustment Tolerance	Auto

System - Adjust Volume

Position	Isocenter
Orientation	Transversal
Rotation	0.00 deg
A >> P	263 mm
R >> L	350 mm
F >> H	350 mm
Reset	Off

System - Tx/Rx

Frequency 1H	123.219268 MHz
Correction factor	1
Gain	High
Img. Scale Cor.	1.000
Reset	Off
? Ref. amplitude 1H	0.000 V

Physio - Signal1

1st Signal/Mode	None
TR	7200.0 ms
Concatenations	1

Physio - Cardiac

Magn. preparation	None
Fat suppr.	None
Dark blood	Off
FoV read	200 mm
FoV phase	100.0 %
Phase resolution	85 %
Trajectory	Cartesian

Physio - PACE

Resp. control	Off
Concatenations	1

Inline - Common

Subtract	Off
Measurements	1
StdDev	Off
Save original images	On

Inline - MIP

MIP-Sag	Off
MIP-Cor	Off
MIP-Tra	Off
MIP-Time	Off
Save original images	On

Inline - Composing

Inline Composing	Off
Distortion Corr.	Off

Sequence - Part 1

Introduction	On
Dimension	2D
Compensate T2 decay	Off
Reduce Motion Sens.	On
Contrasts	1
Flow comp.	No
Multi-slice mode	Interleaved
Free echo spacing	Off
Echo spacing	11.2 ms
Bandwidth	200 Hz/Px

Sequence - Part 2

Define	Turbo factor
Echo trains per slice	11
Phase correction	Automatic
Acoustic noise reduction	None
RF pulse type	Low SAR
Gradient mode	Normal
Hyperecho	Off
WARP	Off
Red. EC sensitivity	Off
Turbo factor	25

Sequence - Assistant

Mode	Off
Allowed delay	30 s

\\Vanha Verio\RESEARCH\Prostata\FLUCIPRO\diff_tra_b0_100_200_350_500

TA: 5:10 PM: REF Voxel size: 2.0×2.0×3.0 mmPAT: 2 Rel. SNR: 1.00 : epse

Properties

Prio recon	Off
Load images to viewer	On
Inline movie	Off
Auto store images	On
Load images to stamp segments	Off
Load images to graphic segments	Off
Auto open inline display	Off
Auto close inline display	Off
Start measurement without further preparation	Off
Wait for user to start	Off
Start measurements	Single measurement

Routine

Slice group	1
Slices	16
Dist. factor	0 %
Position	L8.4 P30.5 F105.4 mm
Orientation	Transversal
Phase enc. dir.	A >> P
AutoAlign	---
Phase oversampling	25 %
FoV read	260 mm
FoV phase	100.0 %
Slice thickness	3.0 mm
TR	5600 ms
TE	80.0 ms
Averages	4
Concatenations	1
Filter	Raw filter, Prescan Normalize
Coil elements	BO1-3;SP3

Contrast - Common

TR	5600 ms
TE	80.0 ms
MTC	Off
Magn. preparation	None
Fat suppr.	SPAIR
Fat sat. mode	Strong

Contrast - Dynamic

Averages	4
Averaging mode	Long term
Reconstruction	Magnitude
Measurements	1
Delay in TR	0 ms

Resolution - Common

FoV read	260 mm
FoV phase	100.0 %
Slice thickness	3.0 mm
Base resolution	128
Phase resolution	100 %
Phase partial Fourier	6/8
Interpolation	Off

Resolution - iPAT

Accel. mode	GRAPPA
Accel. factor PE	2

Resolution - iPAT

Ref. lines PE	24
Reference scan mode	EPI/separate

Resolution - Filter Image

Distortion Corr.	Off
Prescan Normalize	On
Dynamic Field Corr.	Off

Resolution - Filter Rawdata

Raw filter	On
Elliptical filter	Off

Geometry - Common

Slice group	1
Slices	16
Dist. factor	0 %
Position	L8.4 P30.5 F105.4 mm
Orientation	Transversal
Phase enc. dir.	A >> P
FoV read	260 mm
FoV phase	100.0 %
Slice thickness	3.0 mm
TR	5600 ms
Multi-slice mode	Interleaved
Series	Interleaved
Concatenations	1

Geometry - AutoAlign

Slice group	1
Position	L8.4 P30.5 F105.4 mm
Orientation	Transversal
Phase enc. dir.	A >> P
AutoAlign	---
Initial Position	Isocenter
L	0.0 mm
P	0.0 mm
F	0.0 mm
Initial Rotation	0.00 deg
Initial Orientation	Transversal

Geometry - Saturation

Sat. region	1
Thickness	80 mm
Position	L0.0 A66.5 H0.0 mm
Orientation	Coronal
Sat. region	2
Thickness	80 mm
Position	L0.0 P125.2 H0.0 mm
Orientation	Coronal
Fat sat. mode	Strong
Special sat.	None

Geometry - Navigator

Geometry - Tim Planning Suite

Set-n-Go Protocol	Off
Table position	F
Table position	102 mm
Inline Composing	Off

System - Miscellaneous

Positioning mode	REF
Table position	F
Table position	102 mm
MSMA	S - C - T
Sagittal	R >> L
Coronal	A >> P
Transversal	F >> H
Coil Combine Mode	Adaptive Combine
Matrix Optimization	Off
Coil Focus	Flat
AutoAlign	---
Coil Select Mode	Default

System - Adjustments

B0 Shim mode	Standard
B1 Shim mode	TrueForm
Adjust with body coil	Off
Confirm freq. adjustment	Off
Assume Dominant Fat	Off
Assume Silicone	Off
Adjustment Tolerance	Auto

System - Adjust Volume

Position	L8.4 P30.5 F105.4 mm
Orientation	Transversal
Rotation	0.00 deg
A >> P	260 mm
R >> L	260 mm
F >> H	48 mm
Reset	Off

System - Tx/Rx

Frequency 1H	123.219268 MHz
Correction factor	1
Gain	High
Img. Scale Cor.	1.000
Reset	Off
? Ref. amplitude 1H	0.000 V

Physio - Signal1

1st Signal/Mode	None
TR	5600 ms
Concatenations	1

Physio - PACE

Resp. control	Off
Concatenations	1

Diff - Neuro

Diffusion mode	3-Scan Trace
Diff. directions	3
Diffusion Scheme	Bipolar
Diff. weightings	5
b-value 1	0 s/mm²
b-value 2	100 s/mm²
b-value 3	200 s/mm²
b-value 4	350 s/mm²
b-value 5	500 s/mm²
b-value 1	4
b-value 2	4
b-value 3	4
b-value 4	4
b-value 5	4
Diff. weighted images	Off

Diff - Neuro

Trace weighted images	On
ADC maps	Off
FA maps	Off
Mosaic	Off
Tensor	Off
Noise level	20

Diff - Body

Diffusion mode	3-Scan Trace
Diff. directions	3
Diffusion Scheme	Bipolar
Diff. weightings	5
b-value 1	0 s/mm²
b-value 2	100 s/mm²
b-value 3	200 s/mm²
b-value 4	350 s/mm²
b-value 5	500 s/mm²
b-value 1	4
b-value 2	4
b-value 3	4
b-value 4	4
b-value 5	4
Diff. weighted images	Off
Trace weighted images	On
ADC maps	Off
Exponential ADC Maps	Off
FA maps	Off
Invert Gray Scale	On
Calculated Image	Off
b-Value >=	0 s/mm²
Noise level	20

Diff - Composing

Inline Composing	Off
Distortion Corr.	Off

Sequence - Part 1

Introduction	On
Optimization	None
Multi-slice mode	Interleaved
Free echo spacing	Off
Echo spacing	0.95 ms
Bandwidth	1184 Hz/Px

Sequence - Part 2

EPI factor	128
RF pulse type	Low SAR
Gradient mode	Fast

\\Vanha Verio\RESEARCH\Prostata\FLUCIPRO\b_1500

TA: 1:37 PM: REF Voxel size: 2.0×2.0×5.0 mmPAT: 2 Rel. SNR: 1.00 : epse

Properties

Prio recon	Off
Load images to viewer	On
Inline movie	Off
Auto store images	On
Load images to stamp segments	Off
Load images to graphic segments	Off
Auto open inline display	Off
Auto close inline display	Off
Start measurement without further preparation	Off
Wait for user to start	Off
Start measurements	Single measurement

Routine

Slice group	1
Slices	10
Dist. factor	0 %
Position	L8.4 P30.5 F3.4 mm
Orientation	Transversal
Phase enc. dir.	A >> P
AutoAlign	---
Phase oversampling	25 %
FoV read	260 mm
FoV phase	100.0 %
Slice thickness	5.0 mm
TR	5000 ms
TE	87.0 ms
Averages	4
Concatenations	1
Filter	Raw filter, Prescan Normalize
Coil elements	SP3

Contrast - Common

TR	5000 ms
TE	87.0 ms
MTC	Off
Magn. preparation	None
Fat suppr.	SPAIR
Fat sat. mode	Strong

Contrast - Dynamic

Averages	4
Averaging mode	Long term
Reconstruction	Magnitude
Measurements	1
Delay in TR	0 ms

Resolution - Common

FoV read	260 mm
FoV phase	100.0 %
Slice thickness	5.0 mm
Base resolution	128
Phase resolution	100 %
Phase partial Fourier	6/8
Interpolation	Off

Resolution - iPAT

Accel. mode	GRAPPA
Accel. factor PE	2

Resolution - iPAT

Ref. lines PE	24
Reference scan mode	EPI/separate

Resolution - Filter Image

Distortion Corr.	Off
Prescan Normalize	On
Dynamic Field Corr.	Off

Resolution - Filter Rawdata

Raw filter	On
Elliptical filter	Off

Geometry - Common

Slice group	1
Slices	10
Dist. factor	0 %
Position	L8.4 P30.5 F3.4 mm
Orientation	Transversal
Phase enc. dir.	A >> P
FoV read	260 mm
FoV phase	100.0 %
Slice thickness	5.0 mm
TR	5000 ms
Multi-slice mode	Interleaved
Series	Interleaved
Concatenations	1

Geometry - AutoAlign

Slice group	1
Position	L8.4 P30.5 F3.4 mm
Orientation	Transversal
Phase enc. dir.	A >> P
AutoAlign	---
Initial Position	Isocenter
L	0.0 mm
P	0.0 mm
H	0.0 mm
Initial Rotation	0.00 deg
Initial Orientation	Transversal

Geometry - Saturation

Sat. region	1
Thickness	80 mm
Position	L0.0 A66.5 H102.0 mm
Orientation	Coronal
Sat. region	2
Thickness	80 mm
Position	L0.0 P125.2 H102.0 mm
Orientation	Coronal
Fat sat. mode	Strong
Special sat.	None

Geometry - Navigator

Geometry - Tim Planning Suite

Set-n-Go Protocol	Off
Table position	H
Table position	0 mm
Inline Composing	Off

System - Miscellaneous

Positioning mode	REF
Table position	H
Table position	0 mm
MSMA	S - C - T
Sagittal	R >> L
Coronal	A >> P
Transversal	F >> H
Coil Combine Mode	Adaptive Combine
Matrix Optimization	Off
Coil Focus	Flat
AutoAlign	---
Coil Select Mode	Default

System - Adjustments

B0 Shim mode	Standard
B1 Shim mode	TrueForm
Adjust with body coil	Off
Confirm freq. adjustment	Off
Assume Dominant Fat	Off
Assume Silicone	Off
Adjustment Tolerance	Auto

System - Adjust Volume

Position	L8.4 P30.5 F3.4 mm
Orientation	Transversal
Rotation	0.00 deg
A >> P	260 mm
R >> L	260 mm
F >> H	50 mm
Reset	Off

System - Tx/Rx

Frequency 1H	123.219268 MHz
Correction factor	1
Gain	High
Img. Scale Cor.	1.000
Reset	Off
? Ref. amplitude 1H	0.000 V

Physio - Signal1

1st Signal/Mode	None
TR	5000 ms
Concatenations	1

Physio - PACE

Resp. control	Off
Concatenations	1

Diff - Neuro

Diffusion mode	3-Scan Trace
Diff. directions	3
Diffusion Scheme	Bipolar
Diff. weightings	2
b-value 1	0 s/mm²
b-value 2	1500 s/mm²
b-value 1	4
b-value 2	4
Diff. weighted images	Off
Trace weighted images	On
ADC maps	Off
FA maps	Off
Mosaic	Off
Tensor	Off
Noise level	20

Diff - Body

Diffusion mode	3-Scan Trace
Diff. directions	3
Diffusion Scheme	Bipolar
Diff. weightings	2
b-value 1	0 s/mm²
b-value 2	1500 s/mm²
b-value 1	4
b-value 2	4
Diff. weighted images	Off
Trace weighted images	On
ADC maps	Off
Exponential ADC Maps	Off
FA maps	Off
Invert Gray Scale	On
Calculated Image	Off
b-Value >=	0 s/mm²
Noise level	20

Diff - Composing

Inline Composing	Off
Distortion Corr.	Off

Sequence - Part 1

Introduction	On
Optimization	None
Multi-slice mode	Interleaved
Free echo spacing	Off
Echo spacing	0.7 ms
Bandwidth	1628 Hz/Px

Sequence - Part 2

EPI factor	128
RF pulse type	Low SAR
Gradient mode	Fast

\\Vanha Verio\RESEARCH\Prostata\FLUCIPRO\b_2000
TA: 1:37 PM: REF Voxel size: 2.0×2.0×5.0 mmPAT: 2 Rel. SNR: 1.00 : epse

Properties

Prio recon	Off
Load images to viewer	On
Inline movie	Off
Auto store images	On
Load images to stamp segments	Off
Load images to graphic segments	Off
Auto open inline display	Off
Auto close inline display	Off
Start measurement without further preparation	Off
Wait for user to start	Off
Start measurements	Single measurement

Routine

Slice group	1
Slices	10
Dist. factor	0 %
Position	L8.4 P30.5 F3.4 mm
Orientation	Transversal
Phase enc. dir.	A >> P
AutoAlign	---
Phase oversampling	25 %
FoV read	260 mm
FoV phase	100.0 %
Slice thickness	5.0 mm
TR	5000 ms
TE	87.0 ms
Averages	4
Concatenations	1
Filter	Raw filter, Prescan Normalize
Coil elements	SP3

Contrast - Common

TR	5000 ms
TE	87.0 ms
MTC	Off
Magn. preparation	None
Fat suppr.	SPAIR
Fat sat. mode	Strong

Contrast - Dynamic

Averages	4
Averaging mode	Long term
Reconstruction	Magnitude
Measurements	1
Delay in TR	0 ms

Resolution - Common

FoV read	260 mm
FoV phase	100.0 %
Slice thickness	5.0 mm
Base resolution	128
Phase resolution	100 %
Phase partial Fourier	6/8
Interpolation	Off

Resolution - iPAT

Accel. mode	GRAPPA
Accel. factor PE	2

Resolution - iPAT

Ref. lines PE	24
Reference scan mode	EPI/separate

Resolution - Filter Image

Distortion Corr.	Off
Prescan Normalize	On
Dynamic Field Corr.	Off

Resolution - Filter Rawdata

Raw filter	On
Elliptical filter	Off

Geometry - Common

Slice group	1
Slices	10
Dist. factor	0 %
Position	L8.4 P30.5 F3.4 mm
Orientation	Transversal
Phase enc. dir.	A >> P
FoV read	260 mm
FoV phase	100.0 %
Slice thickness	5.0 mm
TR	5000 ms
Multi-slice mode	Interleaved
Series	Interleaved
Concatenations	1

Geometry - AutoAlign

Slice group	1
Position	L8.4 P30.5 F3.4 mm
Orientation	Transversal
Phase enc. dir.	A >> P
AutoAlign	---
Initial Position	Isocenter
L	0.0 mm
P	0.0 mm
H	0.0 mm
Initial Rotation	0.00 deg
Initial Orientation	Transversal

Geometry - Saturation

Sat. region	1
Thickness	80 mm
Position	L0.0 A66.5 H102.0 mm
Orientation	Coronal
Sat. region	2
Thickness	80 mm
Position	L0.0 P125.2 H102.0 mm
Orientation	Coronal
Fat sat. mode	Strong
Special sat.	None

Geometry - Navigator

Geometry - Tim Planning Suite

Set-n-Go Protocol	Off
Table position	H
Table position	0 mm
Inline Composing	Off

System - Miscellaneous

Positioning mode	REF
Table position	H
Table position	0 mm
MSMA	S - C - T
Sagittal	R >> L
Coronal	A >> P
Transversal	F >> H
Coil Combine Mode	Adaptive Combine
Matrix Optimization	Off
Coil Focus	Flat
AutoAlign	---
Coil Select Mode	Default

System - Adjustments

B0 Shim mode	Standard
B1 Shim mode	TrueForm
Adjust with body coil	Off
Confirm freq. adjustment	Off
Assume Dominant Fat	Off
Assume Silicone	Off
Adjustment Tolerance	Auto

System - Adjust Volume

Position	L8.4 P30.5 F3.4 mm
Orientation	Transversal
Rotation	0.00 deg
A >> P	260 mm
R >> L	260 mm
F >> H	50 mm
Reset	Off

System - Tx/Rx

Frequency 1H	123.219268 MHz
Correction factor	1
Gain	High
Img. Scale Cor.	1.000
Reset	Off
? Ref. amplitude 1H	0.000 V

Physio - Signal1

1st Signal/Mode	None
TR	5000 ms
Concatenations	1

Physio - PACE

Resp. control	Off
Concatenations	1

Diff - Neuro

Diffusion mode	3-Scan Trace
Diff. directions	3
Diffusion Scheme	Bipolar
Diff. weightings	2
b-value 1	0 s/mm²
b-value 2	2000 s/mm²
b-value 1	4
b-value 2	4
Diff. weighted images	Off
Trace weighted images	On
ADC maps	Off
FA maps	Off
Mosaic	Off
Tensor	Off
Noise level	20

Diff - Body

Diffusion mode	3-Scan Trace
Diff. directions	3
Diffusion Scheme	Bipolar
Diff. weightings	2
b-value 1	0 s/mm²
b-value 2	2000 s/mm²
b-value 1	4
b-value 2	4
Diff. weighted images	Off
Trace weighted images	On
ADC maps	Off
Exponential ADC Maps	Off
FA maps	Off
Invert Gray Scale	On
Calculated Image	Off
b-Value >=	0 s/mm²
Noise level	20

Diff - Composing

Inline Composing	Off
Distortion Corr.	Off

Sequence - Part 1

Introduction	On
Optimization	None
Multi-slice mode	Interleaved
Free echo spacing	Off
Echo spacing	0.7 ms
Bandwidth	1628 Hz/Px

Sequence - Part 2

EPI factor	128
RF pulse type	Low SAR
Gradient mode	Fast

\\Vanha Verio\RESEARCH\Prostata\FLUCIPRO\csi3d_P_12x12x12_8x8x8_I_NA4

TA: 12:36 PM: REF Voxel size: 8.0×8.0×8.0 mmRel. SNR: 1.00 : csi_se

Properties

Prio recon	Off
Load images to viewer	On
Inline movie	Off
Auto store images	On
Load images to stamp segments	Off
Load images to graphic segments	Off
Auto open inline display	Off
Auto close inline display	Off
Start measurement without further preparation	Off
Wait for user to start	Off
Start measurements	Single measurement

Routine

Position	L10.6 P15.1 F1.3 mm
Orientation	Transversal
Rotation	0 deg
Slabs	1
VoI A >> P	36 mm
VoI R >> L	48 mm
VoI F >> H	36 mm
FoV A >> P	96 mm
FoV R >> L	96 mm
FoV F >> H	96 mm
TR	750 ms
TE	140 ms
Averages	6
Filter	Prescan Normalize, Hamming
Coil elements	BO1-3;SP3,4

Contrast

TR	750 ms
TE	140 ms
Averages	6
Averaging mode	Long term
Flip angle	90 deg
Application	Prostate
Water suppr.	None
Spectral suppr.	Lipid + Water suppr.
Lipid suppr. BW	1.00 ppm
Lipid s. delta pos.	-3.40 ppm
Water s. BW	1.00 ppm
Water s. delta pos.	0.00 ppm
Measurements	1

Resolution - Common

FoV R >> L	96 mm
FoV A >> P	96 mm
FoV F >> H	96 mm
Scan res. R >> L	12
Scan res. A >> P	12
Scan res. F >> H	12
Interpol. res. R >> L	16
Interpol. res. A >> P	16
Interpol. res. F >> H	16
Hamming	On
Width	100
Prescan Normalize	On
Vector size	512

Geometry - Common

Position	L10.6 P15.1 F1.3 mm
Orientation	Transversal
Rotation	0 deg
FoV R >> L	96 mm
FoV A >> P	96 mm
FoV F >> H	96 mm
VoI R >> L	48 mm
VoI A >> P	36 mm
VoI F >> H	36 mm
Fully excited VoI	Off
Sat. region	1
Thickness	60 mm
Position	R46.9 P46.9 H0.0 mm
Orientation	C > S45.0
Sat. delta frequ.	-3.40 ppm
Sat. region	2
Thickness	60 mm
Position	L51.4 P55.9 H0.0 mm
Orientation	C > S-42.6
Sat. delta frequ.	-3.40 ppm
Sat. region	3
Thickness	60 mm
Position	L0.5 P69.7 H0.0 mm
Orientation	C > S-0.4
Sat. delta frequ.	-3.40 ppm
Sat. region	4
Thickness	60 mm
Position	R29.5 A29.5 H0.0 mm
Orientation	C > S-45.0
Sat. delta frequ.	-3.40 ppm
Sat. region	5
Thickness	60 mm
Position	L37.0 A37.2 H0.0 mm
Orientation	C > S44.9
Sat. delta frequ.	-3.40 ppm
Sat. region	6
Thickness	60 mm
Position	R18.9 P8.1 F84.1 mm
Orientation	Transversal
Sat. delta frequ.	-3.40 ppm
Sat. region	7
Thickness	60 mm
Position	R18.9 P8.1 H56.5 mm
Orientation	Transversal
Sat. delta frequ.	-3.40 ppm

Geometry - AutoAlign

Slab group	1
Position	L10.6 P15.1 F1.3 mm
Orientation	Transversal
Phase enc. dir.	A >> P
AutoAlign	---
Initial Position	Isocenter
L	0.0 mm
P	0.0 mm
H	0.0 mm
Initial Rotation	0.00 deg
Initial Orientation	Transversal

System - Miscellaneous

Positioning mode	REF
Table position	H
Table position	4 mm
MSMA	S - C - T
Sagittal	R >> L
Coronal	A >> P
Transversal	F >> H
Save uncombined	Off
AutoAlign	---
Coil Select Mode	Default

System - Adjustments

B0 Shim mode	Advanced
B1 Shim mode	TrueForm
Adj. water suppr.	On
Adjust with body coil	Off
Confirm freq. adjustment	On
Only after freq. change	Off
Assume Dominant Fat	Off
Assume Silicone	Off
Adjustment Tolerance	Auto

System - Adjust Volume

Position	L10.6 P15.1 F1.3 mm
Orientation	Transversal
Rotation	0.00 deg
A >> P	36 mm
R >> L	48 mm
F >> H	36 mm
Reset	Off

System - Tx/Rx

Frequency 1H	123.219268 MHz
Gain	High
Img. Scale Cor.	1.000
Reset	Off
? Ref. amplitude 1H	0.000 V

Sequence - Common

Preparation scans	4
Dimension	3D
Delta frequency	-1.80 ppm
Phase encoding	Weighted
Bandwidth	1300 Hz
Acquisition duration	393 ms
Remove oversampling	On

\\Vanha Verio\RESEARCH\Prostata\FLUCIPRO\d_16b_equally
TA: 11:07 PM: REF Voxel size: 2.0×2.0×5.0 mmPAT: 2 Rel. SNR: 1.00 : epse

Properties

Prio recon	Off
Load images to viewer	On
Inline movie	Off
Auto store images	On
Load images to stamp segments	Off
Load images to graphic segments	Off
Auto open inline display	Off
Auto close inline display	Off
Start measurement without further preparation	Off
Wait for user to start	Off
Start measurements	Single measurement

Routine

Slice group	1
Slices	14
Dist. factor	0 %
Position	L8.4 P30.5 F105.4 mm
Orientation	Transversal
Phase enc. dir.	A >> P
AutoAlign	---
Phase oversampling	25 %
FoV read	260 mm
FoV phase	100.0 %
Slice thickness	5.0 mm
TR	7000 ms
TE	87.0 ms
Averages	2
Concatenations	1
Filter	Raw filter, Prescan Normalize
Coil elements	BO1-3;SP3

Contrast - Common

TR	7000 ms
TE	87.0 ms
MTC	Off
Magn. preparation	None
Fat suppr.	SPAIR
Fat sat. mode	Strong

Contrast - Dynamic

Averages	2
Averaging mode	Long term
Reconstruction	Magnitude
Measurements	1
Delay in TR	0 ms

Resolution - Common

FoV read	260 mm
FoV phase	100.0 %
Slice thickness	5.0 mm
Base resolution	128
Phase resolution	100 %
Phase partial Fourier	6/8
Interpolation	Off

Resolution - iPAT

Accel. mode	GRAPPA
Accel. factor PE	2

Resolution - iPAT

Ref. lines PE	24
Reference scan mode	EPI/separate

Resolution - Filter Image

Distortion Corr.	Off
Prescan Normalize	On
Dynamic Field Corr.	Off

Resolution - Filter Rawdata

Raw filter	On
Elliptical filter	Off

Geometry - Common

Slice group	1
Slices	14
Dist. factor	0 %
Position	L8.4 P30.5 F105.4 mm
Orientation	Transversal
Phase enc. dir.	A >> P
FoV read	260 mm
FoV phase	100.0 %
Slice thickness	5.0 mm
TR	7000 ms
Multi-slice mode	Interleaved
Series	Interleaved
Concatenations	1

Geometry - AutoAlign

Slice group	1
Position	L8.4 P30.5 F105.4 mm
Orientation	Transversal
Phase enc. dir.	A >> P
AutoAlign	---
Initial Position	Isocenter
L	0.0 mm
P	0.0 mm
F	0.0 mm
Initial Rotation	0.00 deg
Initial Orientation	Transversal

Geometry - Saturation

Sat. region	1
Thickness	80 mm
Position	L0.0 A66.5 H0.0 mm
Orientation	Coronal
Sat. region	2
Thickness	80 mm
Position	L0.0 P125.2 H0.0 mm
Orientation	Coronal
Fat sat. mode	Strong
Special sat.	None

Geometry - Navigator

Geometry - Tim Planning Suite

Set-n-Go Protocol	Off
Table position	F
Table position	102 mm
Inline Composing	Off

System - Miscellaneous

Positioning mode	REF
Table position	F
Table position	102 mm
MSMA	S - C - T
Sagittal	R >> L
Coronal	A >> P
Transversal	F >> H
Coil Combine Mode	Adaptive Combine
Matrix Optimization	Off
Coil Focus	Flat
AutoAlign	---
Coil Select Mode	Default

System - Adjustments

B0 Shim mode	Standard
B1 Shim mode	TrueForm
Adjust with body coil	Off
Confirm freq. adjustment	Off
Assume Dominant Fat	Off
Assume Silicone	Off
Adjustment Tolerance	Auto

System - Adjust Volume

Position	L8.4 P30.5 F105.4 mm
Orientation	Transversal
Rotation	0.00 deg
A >> P	260 mm
R >> L	260 mm
F >> H	70 mm
Reset	Off

System - Tx/Rx

Frequency 1H	123.219268 MHz
Correction factor	1
Gain	High
Img. Scale Cor.	1.000
Reset	Off
? Ref. amplitude 1H	0.000 V

Physio - Signal1

1st Signal/Mode	None
TR	7000 ms
Concatenations	1

Physio - PACE

Resp. control	Off
Concatenations	1

Diff - Neuro

Diffusion mode	3-Scan Trace
Diff. directions	3
Diffusion Scheme	Bipolar
Diff. weightings	16
b-value 1	0 s/mm²
b-value 2	50 s/mm²
b-value 3	100 s/mm²
b-value 4	200 s/mm²
b-value 5	350 s/mm²
b-value 6	500 s/mm²
b-value 7	650 s/mm²
b-value 8	800 s/mm²
b-value 9	950 s/mm²
b-value 10	1100 s/mm²
b-value 11	1250 s/mm²
b-value 12	1400 s/mm²
b-value 13	1550 s/mm²
b-value 14	1700 s/mm²
b-value 15	1850 s/mm²
b-value 16	2000 s/mm²
b-value 1	2
b-value 2	2
b-value 3	2
b-value 4	2
b-value 5	2
b-value 6	2
b-value 7	2
b-value 8	2
b-value 9	2
b-value 10	2
b-value 11	2
b-value 12	2
b-value 13	2
b-value 14	2
b-value 15	2
b-value 16	2
Diff. weighted images	Off
Trace weighted images	On
ADC maps	Off
FA maps	Off
Mosaic	Off
Tensor	Off
Noise level	20

Diff - Body

Diffusion mode	3-Scan Trace
Diff. directions	3
Diffusion Scheme	Bipolar
Diff. weightings	16
b-value 1	0 s/mm²
b-value 2	50 s/mm²
b-value 3	100 s/mm²
b-value 4	200 s/mm²
b-value 5	350 s/mm²
b-value 6	500 s/mm²
b-value 7	650 s/mm²
b-value 8	800 s/mm²
b-value 9	950 s/mm²
b-value 10	1100 s/mm²
b-value 11	1250 s/mm²
b-value 12	1400 s/mm²
b-value 13	1550 s/mm²
b-value 14	1700 s/mm²
b-value 15	1850 s/mm²
b-value 16	2000 s/mm²
b-value 1	2
b-value 2	2
b-value 3	2
b-value 4	2
b-value 5	2
b-value 6	2
b-value 7	2
b-value 8	2
b-value 9	2
b-value 10	2
b-value 11	2
b-value 12	2
b-value 13	2
b-value 14	2

Diff - Body

b-value 15	2
b-value 16	2
Diff. weighted images	Off
Trace weighted images	On
ADC maps	Off
Exponential ADC Maps	Off
FA maps	Off
Invert Gray Scale	On
Calculated Image	Off
b-Value >=	0 s/mm²
Noise level	20

Diff - Composing

Inline Composing	Off
Distortion Corr.	Off

Sequence - Part 1

Introduction	On
Optimization	None
Multi-slice mode	Interleaved
Free echo spacing	Off
Echo spacing	0.7 ms
Bandwidth	1628 Hz/Px

Sequence - Part 2

EPI factor	128
RF pulse type	Low SAR
Gradient mode	Fast

\\Vanha Verio\RESEARCH\Prostata\FLUCIPRO\t1_vibe_tra_FA2
TA: 6.6 s PM: REF Voxel size: 1.3×1.3×3.0 mmPAT: 2 Rel. SNR: 1.00 : fl

Properties

Prio recon	Off
Load images to viewer	Off
Inline movie	Off
Auto store images	On
Load images to stamp segments	Off
Load images to graphic segments	Off
Auto open inline display	Off
Auto close inline display	Off
Start measurement without further preparation	Off
Wait for user to start	Off
Start measurements	Single measurement

Routine

Slab group	1
Slabs	1
Dist. factor	20 %
Position	L40.0 P21.4 F99.2 mm
Orientation	Transversal
Phase enc. dir.	R >> L
AutoAlign	---
Phase oversampling	50 %
Slice oversampling	25.0 %
Slices per slab	16
FoV read	240 mm
FoV phase	100.0 %
Slice thickness	3.0 mm
TR	5.43 ms
TE	1.87 ms
Averages	1
Concatenations	1
Filter	Prescan Normalize
Coil elements	BO1-3;SP6

Contrast - Common

TR	5.43 ms
TE	1.87 ms
Flip angle	2.0 deg
Fat suppr.	None
Water suppr.	None
Dixon	Off

Contrast - Dynamic

Averages	1
Averaging mode	Short term
Reconstruction	Magnitude
Measurements	1
Multiple series	Each measurement

Resolution - Common

FoV read	240 mm
FoV phase	100.0 %
Slice thickness	3.0 mm
Base resolution	192
Phase resolution	100 %
Slice resolution	70 %
Phase partial Fourier	6/8
Slice partial Fourier	6/8
Trajectory	Cartesian
View sharing	Off

Resolution - Common

Interpolation	Off

Resolution - iPAT

PAT mode	GRAPPA
Accel. factor PE	2
Ref. lines PE	24
Accel. factor 3D	1
Reference scan mode	Integrated

Resolution - Filter Image

Image Filter	Off
Distortion Corr.	Off
Prescan Normalize	On
Unfiltered images	Off
Normalize	Off
B1 filter	Off

Resolution - Filter Rawdata

Raw filter	Off
Elliptical filter	Off
POCS	Off

Geometry - Common

Slab group	1
Slabs	1
Dist. factor	20 %
Position	L40.0 P21.4 F99.2 mm
Orientation	Transversal
Phase enc. dir.	R >> L
Slice oversampling	25.0 %
Slices per slab	16
FoV read	240 mm
FoV phase	100.0 %
Slice thickness	3.0 mm
TR	5.43 ms
Multi-slice mode	Sequential
Series	Ascending
Concatenations	1

Geometry - AutoAlign

Slab group	1
Position	L40.0 P21.4 F99.2 mm
Orientation	Transversal
Phase enc. dir.	R >> L
AutoAlign	---
Initial Position	Isocenter
L	0.0 mm
P	0.0 mm
F	0.0 mm
Initial Rotation	0.00 deg
Initial Orientation	Transversal

Geometry - Saturation

Fat suppr.	None
Water suppr.	None
Dixon	Off
Special sat.	None

Geometry - Tim Planning Suite

Set-n-Go Protocol	Off

Geometry - Tim Planning Suite

Table position	F
Table position	98 mm
Inline Composing	Off

System - Miscellaneous

Positioning mode	REF
Table position	F
Table position	98 mm
MSMA	S - C - T
Sagittal	R >> L
Coronal	A >> P
Transversal	F >> H
Coil Combine Mode	Adaptive Combine
Save uncombined	Off
Matrix Optimization	Off
Coil Focus	Flat
AutoAlign	---
Coil Select Mode	Default

System - Adjustments

B0 Shim mode	Standard
B1 Shim mode	TrueForm
Adjust with body coil	Off
Confirm freq. adjustment	Off
Assume Dominant Fat	Off
Assume Silicone	Off
Adjustment Tolerance	Auto

System - Adjust Volume

Position	L40.0 P21.4 F99.2 mm
Orientation	Transversal
Rotation	90.00 deg
R >> L	240 mm
A >> P	240 mm
F >> H	48 mm
Reset	Off

System - Tx/Rx

Frequency 1H	123.219268 MHz
Correction factor	1
Gain	Low
Img. Scale Cor.	1.000
Reset	Off
? Ref. amplitude 1H	0.000 V

Physio - PACE

Resp. control	Off
Concatenations	1

Inline - Common

View sharing	Off
Flip angle	2.0 deg
Measurements	1
Burn time-to-center	Off
Temporal interpolation	1
3D centric reordering	Off
Time to center	2.7 s

Inline - Inline

Subtract	Off
Measurements	1
StdDev	Off
Liver registration	Off

Inline - Inline

Save original images	On

Inline - MIP

MIP-Sag	Off
MIP-Cor	Off
MIP-Tra	Off
MIP-Time	Off
Save original images	On

Inline - Soft Tissue

Wash - In	Off
Wash - Out	Off
TTP	Off
PEI	Off
MIP - time	Off
Measurements	1

Inline - Composing

Inline Composing	Off
Distortion Corr.	Off

Inline - MapIt

Save original images	On
MapIt	None
Flip angle	2.0 deg
Measurements	1
Contrasts	1
TR	5.43 ms
TE	1.87 ms

Sequence - Part 1

Introduction	Off
Dimension	3D
Elliptical scanning	On
Asymmetric echo	Weak
Contrasts	1
Optimization	Min. TE
Multi-slice mode	Sequential
Bandwidth	260 Hz/Px

Sequence - Part 2

RF pulse type	Normal
Gradient mode	Fast
Excitation	Slab-sel.
RF spoiling	On
Incr. Gradient spoiling	Off

Sequence - Assistant

Mode	Off
Allowed delay	0 s

\\Vanha Verio\RESEARCH\Prostata\FLUCIPRO\t1_vibe_tra_FA5
TA: 6.6 s PM: REF Voxel size: 1.3×1.3×3.0 mmPAT: 2 Rel. SNR: 1.00 : fl

Properties

Prio recon	Off
Load images to viewer	Off
Inline movie	Off
Auto store images	On
Load images to stamp segments	Off
Load images to graphic segments	Off
Auto open inline display	Off
Auto close inline display	Off
Start measurement without further preparation	Off
Wait for user to start	Off
Start measurements	Single measurement

Routine

Slab group	1
Slabs	1
Dist. factor	20 %
Position	L40.0 P21.4 F99.2 mm
Orientation	Transversal
Phase enc. dir.	R >> L
AutoAlign	---
Phase oversampling	50 %
Slice oversampling	25.0 %
Slices per slab	16
FoV read	240 mm
FoV phase	100.0 %
Slice thickness	3.0 mm
TR	5.43 ms
TE	1.87 ms
Averages	1
Concatenations	1
Filter	Prescan Normalize
Coil elements	BO1-3;SP6

Contrast - Common

TR	5.43 ms
TE	1.87 ms
Flip angle	5.0 deg
Fat suppr.	None
Water suppr.	None
Dixon	Off

Contrast - Dynamic

Averages	1
Averaging mode	Short term
Reconstruction	Magnitude
Measurements	1
Multiple series	Each measurement

Resolution - Common

FoV read	240 mm
FoV phase	100.0 %
Slice thickness	3.0 mm
Base resolution	192
Phase resolution	100 %
Slice resolution	70 %
Phase partial Fourier	6/8
Slice partial Fourier	6/8
Trajectory	Cartesian
View sharing	Off

Resolution - Common

Interpolation	Off

Resolution - iPAT

PAT mode	GRAPPA
Accel. factor PE	2
Ref. lines PE	24
Accel. factor 3D	1
Reference scan mode	Integrated

Resolution - Filter Image

Image Filter	Off
Distortion Corr.	Off
Prescan Normalize	On
Unfiltered images	Off
Normalize	Off
B1 filter	Off

Resolution - Filter Rawdata

Raw filter	Off
Elliptical filter	Off
POCS	Off

Geometry - Common

Slab group	1
Slabs	1
Dist. factor	20 %
Position	L40.0 P21.4 F99.2 mm
Orientation	Transversal
Phase enc. dir.	R >> L
Slice oversampling	25.0 %
Slices per slab	16
FoV read	240 mm
FoV phase	100.0 %
Slice thickness	3.0 mm
TR	5.43 ms
Multi-slice mode	Sequential
Series	Ascending
Concatenations	1

Geometry - AutoAlign

Slab group	1
Position	L40.0 P21.4 F99.2 mm
Orientation	Transversal
Phase enc. dir.	R >> L
AutoAlign	---
Initial Position	Isocenter
L	0.0 mm
P	0.0 mm
F	0.0 mm
Initial Rotation	0.00 deg
Initial Orientation	Transversal

Geometry - Saturation

Fat suppr.	None
Water suppr.	None
Dixon	Off
Special sat.	None

Geometry - Tim Planning Suite

Set-n-Go Protocol	Off

Geometry - Tim Planning Suite

Table position	F
Table position	98 mm
Inline Composing	Off

System - Miscellaneous

Positioning mode	REF
Table position	F
Table position	98 mm
MSMA	S - C - T
Sagittal	R >> L
Coronal	A >> P
Transversal	F >> H
Coil Combine Mode	Adaptive Combine
Save uncombined	Off
Matrix Optimization	Off
Coil Focus	Flat
AutoAlign	---
Coil Select Mode	Default

System - Adjustments

B0 Shim mode	Standard
B1 Shim mode	TrueForm
Adjust with body coil	Off
Confirm freq. adjustment	Off
Assume Dominant Fat	Off
Assume Silicone	Off
Adjustment Tolerance	Auto

System - Adjust Volume

Position	L40.0 P21.4 F99.2 mm
Orientation	Transversal
Rotation	90.00 deg
R >> L	240 mm
A >> P	240 mm
F >> H	48 mm
Reset	Off

System - Tx/Rx

Frequency 1H	123.219268 MHz
Correction factor	1
Gain	Low
Img. Scale Cor.	1.000
Reset	Off
? Ref. amplitude 1H	0.000 V

Physio - PACE

Resp. control	Off
Concatenations	1

Inline - Common

View sharing	Off
Flip angle	5.0 deg
Measurements	1
Burn time-to-center	Off
Temporal interpolation	1
3D centric reordering	Off
Time to center	2.7 s

Inline - Inline

Subtract	Off
Measurements	1
StdDev	Off
Liver registration	Off

Inline - Inline

Save original images	On

Inline - MIP

MIP-Sag	Off
MIP-Cor	Off
MIP-Tra	Off
MIP-Time	Off
Save original images	On

Inline - Soft Tissue

Wash - In	Off
Wash - Out	Off
TTP	Off
PEI	Off
MIP - time	Off
Measurements	1

Inline - Composing

Inline Composing	Off
Distortion Corr.	Off

Inline - MapIt

Save original images	On
MapIt	None
Flip angle	5.0 deg
Measurements	1
Contrasts	1
TR	5.43 ms
TE	1.87 ms

Sequence - Part 1

Introduction	Off
Dimension	3D
Elliptical scanning	On
Asymmetric echo	Weak
Contrasts	1
Optimization	Min. TE
Multi-slice mode	Sequential
Bandwidth	260 Hz/Px

Sequence - Part 2

RF pulse type	Normal
Gradient mode	Fast
Excitation	Slab-sel.
RF spoiling	On
Incr. Gradient spoiling	Off

Sequence - Assistant

Mode	Off
Allowed delay	0 s

**\\Vanha Verio\RESEARCH\Prostata\FLUCIPRO\t1_vibe_tra_FA8**
TA: 6.6 s PM: REF Voxel size: 1.3×1.3×3.0 mmPAT: 2 Rel. SNR: 1.00 : fl

Properties

Prio recon	Off
Load images to viewer	Off
Inline movie	Off
Auto store images	On
Load images to stamp segments	Off
Load images to graphic segments	Off
Auto open inline display	Off
Auto close inline display	Off
Start measurement without further preparation	Off
Wait for user to start	Off
Start measurements	Single measurement

Routine

Slab group	1
Slabs	1
Dist. factor	20 %
Position	L40.0 P21.4 F99.2 mm
Orientation	Transversal
Phase enc. dir.	R >> L
AutoAlign	---
Phase oversampling	50 %
Slice oversampling	25.0 %
Slices per slab	16
FoV read	240 mm
FoV phase	100.0 %
Slice thickness	3.0 mm
TR	5.43 ms
TE	1.87 ms
Averages	1
Concatenations	1
Filter	Prescan Normalize
Coil elements	BO1-3;SP6

Contrast - Common

TR	5.43 ms
TE	1.87 ms
Flip angle	8.0 deg
Fat suppr.	None
Water suppr.	None
Dixon	Off

Contrast - Dynamic

Averages	1
Averaging mode	Short term
Reconstruction	Magnitude
Measurements	1
Multiple series	Each measurement

Resolution - Common

FoV read	240 mm
FoV phase	100.0 %
Slice thickness	3.0 mm
Base resolution	192
Phase resolution	100 %
Slice resolution	70 %
Phase partial Fourier	6/8
Slice partial Fourier	6/8
Trajectory	Cartesian
View sharing	Off

Resolution - Common

Interpolation	Off

Resolution - iPAT

PAT mode	GRAPPA
Accel. factor PE	2
Ref. lines PE	24
Accel. factor 3D	1
Reference scan mode	Integrated

Resolution - Filter Image

Image Filter	Off
Distortion Corr.	Off
Prescan Normalize	On
Unfiltered images	Off
Normalize	Off
B1 filter	Off

Resolution - Filter Rawdata

Raw filter	Off
Elliptical filter	Off
POCS	Off

Geometry - Common

Slab group	1
Slabs	1
Dist. factor	20 %
Position	L40.0 P21.4 F99.2 mm
Orientation	Transversal
Phase enc. dir.	R >> L
Slice oversampling	25.0 %
Slices per slab	16
FoV read	240 mm
FoV phase	100.0 %
Slice thickness	3.0 mm
TR	5.43 ms
Multi-slice mode	Sequential
Series	Ascending
Concatenations	1

Geometry - AutoAlign

Slab group	1
Position	L40.0 P21.4 F99.2 mm
Orientation	Transversal
Phase enc. dir.	R >> L
AutoAlign	---
Initial Position	Isocenter
L	0.0 mm
P	0.0 mm
F	0.0 mm
Initial Rotation	0.00 deg
Initial Orientation	Transversal

Geometry - Saturation

Fat suppr.	None
Water suppr.	None
Dixon	Off
Special sat.	None

Geometry - Tim Planning Suite

Set-n-Go Protocol	Off

Geometry - Tim Planning Suite

Table position	F
Table position	98 mm
Inline Composing	Off

System - Miscellaneous

Positioning mode	REF
Table position	F
Table position	98 mm
MSMA	S - C - T
Sagittal	R >> L
Coronal	A >> P
Transversal	F >> H
Coil Combine Mode	Adaptive Combine
Save uncombined	Off
Matrix Optimization	Off
Coil Focus	Flat
AutoAlign	---
Coil Select Mode	Default

System - Adjustments

B0 Shim mode	Standard
B1 Shim mode	TrueForm
Adjust with body coil	Off
Confirm freq. adjustment	Off
Assume Dominant Fat	Off
Assume Silicone	Off
Adjustment Tolerance	Auto

System - Adjust Volume

Position	L40.0 P21.4 F99.2 mm
Orientation	Transversal
Rotation	90.00 deg
R >> L	240 mm
A >> P	240 mm
F >> H	48 mm
Reset	Off

System - Tx/Rx

Frequency 1H	123.219268 MHz
Correction factor	1
Gain	Low
Img. Scale Cor.	1.000
Reset	Off
? Ref. amplitude 1H	0.000 V

Physio - PACE

Resp. control	Off
Concatenations	1

Inline - Common

View sharing	Off
Flip angle	8.0 deg
Measurements	1
Burn time-to-center	Off
Temporal interpolation	1
3D centric reordering	Off
Time to center	2.7 s

Inline - Inline

Subtract	Off
Measurements	1
StdDev	Off
Liver registration	Off

Inline - Inline

Save original images	On

Inline - MIP

MIP-Sag	Off
MIP-Cor	Off
MIP-Tra	Off
MIP-Time	Off
Save original images	On

Inline - Soft Tissue

Wash - In	Off
Wash - Out	Off
TTP	Off
PEI	Off
MIP - time	Off
Measurements	1

Inline - Composing

Inline Composing	Off
Distortion Corr.	Off

Inline - MapIt

Save original images	On
MapIt	None
Flip angle	8.0 deg
Measurements	1
Contrasts	1
TR	5.43 ms
TE	1.87 ms

Sequence - Part 1

Introduction	Off
Dimension	3D
Elliptical scanning	On
Asymmetric echo	Weak
Contrasts	1
Optimization	Min. TE
Multi-slice mode	Sequential
Bandwidth	260 Hz/Px

Sequence - Part 2

RF pulse type	Normal
Gradient mode	Fast
Excitation	Slab-sel.
RF spoiling	On
Incr. Gradient spoiling	Off

Sequence - Assistant

Mode	Off
Allowed delay	0 s

\\Vanha Verio\RESEARCH\Prostata\FLUCIPRO\t1_vibe_tra_FA10
TA: 6.6 s PM: REF Voxel size: 1.3×1.3×3.0 mmPAT: 2 Rel. SNR: 1.00 : fl

Properties

Prio recon	Off
Load images to viewer	Off
Inline movie	Off
Auto store images	On
Load images to stamp segments	Off
Load images to graphic segments	Off
Auto open inline display	Off
Auto close inline display	Off
Start measurement without further preparation	Off
Wait for user to start	Off
Start measurements	Single measurement

Routine

Slab group	1
Slabs	1
Dist. factor	20 %
Position	L40.0 P21.4 F99.2 mm
Orientation	Transversal
Phase enc. dir.	R >> L
AutoAlign	---
Phase oversampling	50 %
Slice oversampling	25.0 %
Slices per slab	16
FoV read	240 mm
FoV phase	100.0 %
Slice thickness	3.0 mm
TR	5.43 ms
TE	1.87 ms
Averages	1
Concatenations	1
Filter	Prescan Normalize
Coil elements	BO1-3;SP6

Contrast - Common

TR	5.43 ms
TE	1.87 ms
Flip angle	10.0 deg
Fat suppr.	None
Water suppr.	None
Dixon	Off

Contrast - Dynamic

Averages	1
Averaging mode	Short term
Reconstruction	Magnitude
Measurements	1
Multiple series	Each measurement

Resolution - Common

FoV read	240 mm
FoV phase	100.0 %
Slice thickness	3.0 mm
Base resolution	192
Phase resolution	100 %
Slice resolution	70 %
Phase partial Fourier	6/8
Slice partial Fourier	6/8
Trajectory	Cartesian
View sharing	Off

Resolution - Common

Interpolation	Off

Resolution - iPAT

PAT mode	GRAPPA
Accel. factor PE	2
Ref. lines PE	24
Accel. factor 3D	1
Reference scan mode	Integrated

Resolution - Filter Image

Image Filter	Off
Distortion Corr.	Off
Prescan Normalize	On
Unfiltered images	Off
Normalize	Off
B1 filter	Off

Resolution - Filter Rawdata

Raw filter	Off
Elliptical filter	Off
POCS	Off

Geometry - Common

Slab group	1
Slabs	1
Dist. factor	20 %
Position	L40.0 P21.4 F99.2 mm
Orientation	Transversal
Phase enc. dir.	R >> L
Slice oversampling	25.0 %
Slices per slab	16
FoV read	240 mm
FoV phase	100.0 %
Slice thickness	3.0 mm
TR	5.43 ms
Multi-slice mode	Sequential
Series	Ascending
Concatenations	1

Geometry - AutoAlign

Slab group	1
Position	L40.0 P21.4 F99.2 mm
Orientation	Transversal
Phase enc. dir.	R >> L
AutoAlign	---
Initial Position	Isocenter
L	0.0 mm
P	0.0 mm
F	0.0 mm
Initial Rotation	0.00 deg
Initial Orientation	Transversal

Geometry - Saturation

Fat suppr.	None
Water suppr.	None
Dixon	Off
Special sat.	None

Geometry - Tim Planning Suite

Set-n-Go Protocol	Off

Geometry - Tim Planning Suite

Table position	F
Table position	98 mm
Inline Composing	Off

System - Miscellaneous

Positioning mode	REF
Table position	F
Table position	98 mm
MSMA	S - C - T
Sagittal	R >> L
Coronal	A >> P
Transversal	F >> H
Coil Combine Mode	Adaptive Combine
Save uncombined	Off
Matrix Optimization	Off
Coil Focus	Flat
AutoAlign	---
Coil Select Mode	Default

System - Adjustments

B0 Shim mode	Standard
B1 Shim mode	TrueForm
Adjust with body coil	Off
Confirm freq. adjustment	Off
Assume Dominant Fat	Off
Assume Silicone	Off
Adjustment Tolerance	Auto

System - Adjust Volume

Position	L40.0 P21.4 F99.2 mm
Orientation	Transversal
Rotation	90.00 deg
R >> L	240 mm
A >> P	240 mm
F >> H	48 mm
Reset	Off

System - Tx/Rx

Frequency 1H	123.219268 MHz
Correction factor	1
Gain	Low
Img. Scale Cor.	1.000
Reset	Off
? Ref. amplitude 1H	0.000 V

Physio - PACE

Resp. control	Off
Concatenations	1

Inline - Common

View sharing	Off
Flip angle	10.0 deg
Measurements	1
Burn time-to-center	Off
Temporal interpolation	1
3D centric reordering	Off
Time to center	2.7 s

Inline - Inline

Subtract	Off
Measurements	1
StdDev	Off
Liver registration	Off

Inline - Inline

Save original images	On

Inline - MIP

MIP-Sag	Off
MIP-Cor	Off
MIP-Tra	Off
MIP-Time	Off
Save original images	On

Inline - Soft Tissue

Wash - In	Off
Wash - Out	Off
TTP	Off
PEI	Off
MIP - time	Off
Measurements	1

Inline - Composing

Inline Composing	Off
Distortion Corr.	Off

Inline - MapIt

Save original images	On
MapIt	None
Flip angle	10.0 deg
Measurements	1
Contrasts	1
TR	5.43 ms
TE	1.87 ms

Sequence - Part 1

Introduction	Off
Dimension	3D
Elliptical scanning	On
Asymmetric echo	Weak
Contrasts	1
Optimization	Min. TE
Multi-slice mode	Sequential
Bandwidth	260 Hz/Px

Sequence - Part 2

RF pulse type	Normal
Gradient mode	Fast
Excitation	Slab-sel.
RF spoiling	On
Incr. Gradient spoiling	Off

Sequence - Assistant

Mode	Off
Allowed delay	0 s

\\Vanha Verio\RESEARCH\Prostata\FLUCIPRO\t1_vibe_tra_FA15
TA: 6.6 s PM: REF Voxel size: 1.3×1.3×3.0 mmPAT: 2 Rel. SNR: 1.00 : fl

Properties

Prio recon	Off
Load images to viewer	Off
Inline movie	Off
Auto store images	On
Load images to stamp segments	Off
Load images to graphic segments	Off
Auto open inline display	Off
Auto close inline display	Off
Start measurement without further preparation	Off
Wait for user to start	Off
Start measurements	Single measurement

Routine

Slab group	1
Slabs	1
Dist. factor	20 %
Position	L40.0 P21.4 F99.2 mm
Orientation	Transversal
Phase enc. dir.	R >> L
AutoAlign	---
Phase oversampling	50 %
Slice oversampling	25.0 %
Slices per slab	16
FoV read	240 mm
FoV phase	100.0 %
Slice thickness	3.0 mm
TR	5.43 ms
TE	1.87 ms
Averages	1
Concatenations	1
Filter	Prescan Normalize
Coil elements	BO1-3;SP6

Contrast - Common

TR	5.43 ms
TE	1.87 ms
Flip angle	15.0 deg
Fat suppr.	None
Water suppr.	None
Dixon	Off

Contrast - Dynamic

Averages	1
Averaging mode	Short term
Reconstruction	Magnitude
Measurements	1
Multiple series	Each measurement

Resolution - Common

FoV read	240 mm
FoV phase	100.0 %
Slice thickness	3.0 mm
Base resolution	192
Phase resolution	100 %
Slice resolution	70 %
Phase partial Fourier	6/8
Slice partial Fourier	6/8
Trajectory	Cartesian
View sharing	Off

Resolution - Common

Interpolation	Off

Resolution - iPAT

PAT mode	GRAPPA
Accel. factor PE	2
Ref. lines PE	24
Accel. factor 3D	1
Reference scan mode	Integrated

Resolution - Filter Image

Image Filter	Off
Distortion Corr.	Off
Prescan Normalize	On
Unfiltered images	Off
Normalize	Off
B1 filter	Off

Resolution - Filter Rawdata

Raw filter	Off
Elliptical filter	Off
POCS	Off

Geometry - Common

Slab group	1
Slabs	1
Dist. factor	20 %
Position	L40.0 P21.4 F99.2 mm
Orientation	Transversal
Phase enc. dir.	R >> L
Slice oversampling	25.0 %
Slices per slab	16
FoV read	240 mm
FoV phase	100.0 %
Slice thickness	3.0 mm
TR	5.43 ms
Multi-slice mode	Sequential
Series	Ascending
Concatenations	1

Geometry - AutoAlign

Slab group	1
Position	L40.0 P21.4 F99.2 mm
Orientation	Transversal
Phase enc. dir.	R >> L
AutoAlign	---
Initial Position	Isocenter
L	0.0 mm
P	0.0 mm
F	0.0 mm
Initial Rotation	0.00 deg
Initial Orientation	Transversal

Geometry - Saturation

Fat suppr.	None
Water suppr.	None
Dixon	Off
Special sat.	None

Geometry - Tim Planning Suite

Set-n-Go Protocol	Off

Geometry - Tim Planning Suite

Table position	F
Table position	98 mm
Inline Composing	Off

System - Miscellaneous

Positioning mode	REF
Table position	F
Table position	98 mm
MSMA	S - C - T
Sagittal	R >> L
Coronal	A >> P
Transversal	F >> H
Coil Combine Mode	Adaptive Combine
Save uncombined	Off
Matrix Optimization	Off
Coil Focus	Flat
AutoAlign	---
Coil Select Mode	Default

System - Adjustments

B0 Shim mode	Standard
B1 Shim mode	TrueForm
Adjust with body coil	Off
Confirm freq. adjustment	Off
Assume Dominant Fat	Off
Assume Silicone	Off
Adjustment Tolerance	Auto

System - Adjust Volume

Position	L40.0 P21.4 F99.2 mm
Orientation	Transversal
Rotation	90.00 deg
R >> L	240 mm
A >> P	240 mm
F >> H	48 mm
Reset	Off

System - Tx/Rx

Frequency 1H	123.219268 MHz
Correction factor	1
Gain	Low
Img. Scale Cor.	1.000
Reset	Off
? Ref. amplitude 1H	0.000 V

Physio - PACE

Resp. control	Off
Concatenations	1

Inline - Common

View sharing	Off
Flip angle	15.0 deg
Measurements	1
Burn time-to-center	Off
Temporal interpolation	1
3D centric reordering	Off
Time to center	2.7 s

Inline - Inline

Subtract	Off
Measurements	1
StdDev	Off
Liver registration	Off

Inline - Inline

Save original images	On

Inline - MIP

MIP-Sag	Off
MIP-Cor	Off
MIP-Tra	Off
MIP-Time	Off
Save original images	On

Inline - Soft Tissue

Wash - In	Off
Wash - Out	Off
TTP	Off
PEI	Off
MIP - time	Off
Measurements	1

Inline - Composing

Inline Composing	Off
Distortion Corr.	Off

Inline - MapIt

Save original images	On
MapIt	None
Flip angle	15.0 deg
Measurements	1
Contrasts	1
TR	5.43 ms
TE	1.87 ms

Sequence - Part 1

Introduction	Off
Dimension	3D
Elliptical scanning	On
Asymmetric echo	Weak
Contrasts	1
Optimization	Min. TE
Multi-slice mode	Sequential
Bandwidth	260 Hz/Px

Sequence - Part 2

RF pulse type	Normal
Gradient mode	Fast
Excitation	Slab-sel.
RF spoiling	On
Incr. Gradient spoiling	Off

Sequence - Assistant

Mode	Off
Allowed delay	0 s

\\Vanha Verio\RESEARCH\Prostata\FLUCIPRO\t1_vibe_tra_FA15_DYN

TA: 6:35 PM: REF Voxel size: 1.3×1.3×3.0 mmPAT: 2 Rel. SNR: 1.00 : fl

Properties

Prio recon	Off
Load images to viewer	Off
Inline movie	Off
Auto store images	On
Load images to stamp segments	Off
Load images to graphic segments	Off
Auto open inline display	Off
Auto close inline display	Off
Start measurement without further preparation	Off
Wait for user to start	On
Start measurements	Single measurement

Routine

Slab group	1
Slabs	1
Dist. factor	20 %
Position	L40.0 P21.4 F99.2 mm
Orientation	Transversal
Phase enc. dir.	R >> L
AutoAlign	---
Phase oversampling	50 %
Slice oversampling	25.0 %
Slices per slab	16
FoV read	240 mm
FoV phase	100.0 %
Slice thickness	3.0 mm
TR	5.43 ms
TE	1.87 ms
Averages	1
Concatenations	1
Filter	Prescan Normalize
Coil elements	BO1-3;SP6

Contrast - Common

TR	5.43 ms
TE	1.87 ms
Flip angle	15.0 deg
Fat suppr.	None
Water suppr.	None
Dixon	Off

Contrast - Dynamic

Averages	1
Averaging mode	Short term
Reconstruction	Magnitude
Measurements	60
Pause after meas. 1	0.0 s
Pause after meas. 2	0.0 s
Pause after meas. 3	0.0 s
Pause after meas. 4	0.0 s
Pause after meas. 5	0.0 s
Pause after meas. 6	0.0 s
Pause after meas. 7	0.0 s
Pause after meas. 8	0.0 s
Pause after meas. 9	0.0 s
Pause after meas. 10	0.0 s
Pause after meas. 11	0.0 s
Pause after meas. 12	0.0 s
Pause after meas. 13	0.0 s

Contrast - Dynamic

Pause after meas. 14	0.0 s
Pause after meas. 15	0.0 s
Pause after meas. 16	0.0 s
Pause after meas. 17	0.0 s
Pause after meas. 18	0.0 s
Pause after meas. 19	0.0 s
Pause after meas. 20	0.0 s
Pause after meas. 21	0.0 s
Pause after meas. 22	0.0 s
Pause after meas. 23	0.0 s
Pause after meas. 24	0.0 s
Pause after meas. 25	0.0 s
Pause after meas. 26	0.0 s
Pause after meas. 27	0.0 s
Pause after meas. 28	0.0 s
Pause after meas. 29	0.0 s
Pause after meas. 30	0.0 s
Pause after meas. 31	0.0 s
Pause after meas. 32	0.0 s
Pause after meas. 33	0.0 s
Pause after meas. 34	0.0 s
Pause after meas. 35	0.0 s
Pause after meas. 36	0.0 s
Pause after meas. 37	0.0 s
Pause after meas. 38	0.0 s
Pause after meas. 39	0.0 s
Pause after meas. 40	0.0 s
Pause after meas. 41	0.0 s
Pause after meas. 42	0.0 s
Pause after meas. 43	0.0 s
Pause after meas. 44	0.0 s
Pause after meas. 45	0.0 s
Pause after meas. 46	0.0 s
Pause after meas. 47	0.0 s
Pause after meas. 48	0.0 s
Pause after meas. 49	0.0 s
Pause after meas. 50	0.0 s
Pause after meas. 51	0.0 s
Pause after meas. 52	0.0 s
Pause after meas. 53	0.0 s
Pause after meas. 54	0.0 s
Pause after meas. 55	0.0 s
Pause after meas. 56	0.0 s
Pause after meas. 57	0.0 s
Pause after meas. 58	0.0 s
Pause after meas. 59	0.0 s
Multiple series	Off

Resolution - Common

FoV read	240 mm
FoV phase	100.0 %
Slice thickness	3.0 mm
Base resolution	192
Phase resolution	100 %
Slice resolution	70 %
Phase partial Fourier	6/8
Slice partial Fourier	6/8
Trajectory	Cartesian
View sharing	Off
Interpolation	Off

Resolution - iPAT

PAT mode	GRAPPA
Accel. factor PE	2
Ref. lines PE	24
Accel. factor 3D	1
Reference scan mode	Integrated

Resolution - Filter Image

Image Filter	Off
Distortion Corr.	Off
Prescan Normalize	On
Unfiltered images	Off
Normalize	Off
B1 filter	Off

Resolution - Filter Rawdata

Raw filter	Off
Elliptical filter	Off
POCS	Off

Geometry - Common

Slab group	1
Slabs	1
Dist. factor	20 %
Position	L40.0 P21.4 F99.2 mm
Orientation	Transversal
Phase enc. dir.	R >> L
Slice oversampling	25.0 %
Slices per slab	16
FoV read	240 mm
FoV phase	100.0 %
Slice thickness	3.0 mm
TR	5.43 ms
Multi-slice mode	Sequential
Series	Ascending
Concatenations	1

Geometry - AutoAlign

Slab group	1
Position	L40.0 P21.4 F99.2 mm
Orientation	Transversal
Phase enc. dir.	R >> L
AutoAlign	---
Initial Position	Isocenter
L	0.0 mm
P	0.0 mm
F	0.0 mm
Initial Rotation	0.00 deg
Initial Orientation	Transversal

Geometry - Saturation

Fat suppr.	None
Water suppr.	None
Dixon	Off
Special sat.	None

Geometry - Tim Planning Suite

Set-n-Go Protocol	Off
Table position	F
Table position	98 mm
Inline Composing	Off

System - Miscellaneous

Positioning mode	REF

System - Miscellaneous

Table position	F
Table position	98 mm
MSMA	S - C - T
Sagittal	R >> L
Coronal	A >> P
Transversal	F >> H
Coil Combine Mode	Adaptive Combine
Save uncombined	Off
Matrix Optimization	Off
Coil Focus	Flat
AutoAlign	---
Coil Select Mode	Default

System - Adjustments

B0 Shim mode	Standard
B1 Shim mode	TrueForm
Adjust with body coil	Off
Confirm freq. adjustment	Off
Assume Dominant Fat	Off
Assume Silicone	Off
Adjustment Tolerance	Auto

System - Adjust Volume

Position	L40.0 P21.4 F99.2 mm
Orientation	Transversal
Rotation	90.00 deg
R >> L	240 mm
A >> P	240 mm
F >> H	48 mm
Reset	Off

System - Tx/Rx

Frequency 1H	123.219268 MHz
Correction factor	1
Gain	Low
Img. Scale Cor.	1.000
Reset	Off
? Ref. amplitude 1H	0.000 V

Physio - PACE

Resp. control	Off
Concatenations	1

Inline - Common

View sharing	Off
Flip angle	15.0 deg
Measurements	60
Pause after meas. 1	0.0 s
Pause after meas. 2	0.0 s
Pause after meas. 3	0.0 s
Pause after meas. 4	0.0 s
Pause after meas. 5	0.0 s
Pause after meas. 6	0.0 s
Pause after meas. 7	0.0 s
Pause after meas. 8	0.0 s
Pause after meas. 9	0.0 s
Pause after meas. 10	0.0 s
Pause after meas. 11	0.0 s
Pause after meas. 12	0.0 s
Pause after meas. 13	0.0 s
Pause after meas. 14	0.0 s
Pause after meas. 15	0.0 s
Pause after meas. 16	0.0 s
Pause after meas. 17	0.0 s

Inline - Common

Pause after meas. 18	0.0 s
Pause after meas. 19	0.0 s
Pause after meas. 20	0.0 s
Pause after meas. 21	0.0 s
Pause after meas. 22	0.0 s
Pause after meas. 23	0.0 s
Pause after meas. 24	0.0 s
Pause after meas. 25	0.0 s
Pause after meas. 26	0.0 s
Pause after meas. 27	0.0 s
Pause after meas. 28	0.0 s
Pause after meas. 29	0.0 s
Pause after meas. 30	0.0 s
Pause after meas. 31	0.0 s
Pause after meas. 32	0.0 s
Pause after meas. 33	0.0 s
Pause after meas. 34	0.0 s
Pause after meas. 35	0.0 s
Pause after meas. 36	0.0 s
Pause after meas. 37	0.0 s
Pause after meas. 38	0.0 s
Pause after meas. 39	0.0 s
Pause after meas. 40	0.0 s
Pause after meas. 41	0.0 s
Pause after meas. 42	0.0 s
Pause after meas. 43	0.0 s
Pause after meas. 44	0.0 s
Pause after meas. 45	0.0 s
Pause after meas. 46	0.0 s
Pause after meas. 47	0.0 s
Pause after meas. 48	0.0 s
Pause after meas. 49	0.0 s
Pause after meas. 50	0.0 s
Pause after meas. 51	0.0 s
Pause after meas. 52	0.0 s
Pause after meas. 53	0.0 s
Pause after meas. 54	0.0 s
Pause after meas. 55	0.0 s
Pause after meas. 56	0.0 s
Pause after meas. 57	0.0 s
Pause after meas. 58	0.0 s
Pause after meas. 59	0.0 s
Burn time-to-center	Off
Temporal interpolation	1
3D centric reordering	Off
Time to center	2.7 s

Inline - Inline

Subtract	Off
Measurements	60
StdDev	Off
Liver registration	Off
Save original images	On

Inline - MIP

MIP-Sag	Off
MIP-Cor	Off
MIP-Tra	Off
MIP-Time	Off
Save original images	On

Inline - Soft Tissue

Wash - In	Off
Wash - Out	Off
TTP	Off
PEI	Off
MIP - time	Off
Measurements	60
Pause after meas. 1	0.0 s
Pause after meas. 2	0.0 s
Pause after meas. 3	0.0 s
Pause after meas. 4	0.0 s
Pause after meas. 5	0.0 s
Pause after meas. 6	0.0 s
Pause after meas. 7	0.0 s
Pause after meas. 8	0.0 s
Pause after meas. 9	0.0 s
Pause after meas. 10	0.0 s
Pause after meas. 11	0.0 s
Pause after meas. 12	0.0 s
Pause after meas. 13	0.0 s
Pause after meas. 14	0.0 s
Pause after meas. 15	0.0 s
Pause after meas. 16	0.0 s
Pause after meas. 17	0.0 s
Pause after meas. 18	0.0 s
Pause after meas. 19	0.0 s
Pause after meas. 20	0.0 s
Pause after meas. 21	0.0 s
Pause after meas. 22	0.0 s
Pause after meas. 23	0.0 s
Pause after meas. 24	0.0 s
Pause after meas. 25	0.0 s
Pause after meas. 26	0.0 s
Pause after meas. 27	0.0 s
Pause after meas. 28	0.0 s
Pause after meas. 29	0.0 s
Pause after meas. 30	0.0 s
Pause after meas. 31	0.0 s
Pause after meas. 32	0.0 s
Pause after meas. 33	0.0 s
Pause after meas. 34	0.0 s
Pause after meas. 35	0.0 s
Pause after meas. 36	0.0 s
Pause after meas. 37	0.0 s
Pause after meas. 38	0.0 s
Pause after meas. 39	0.0 s
Pause after meas. 40	0.0 s
Pause after meas. 41	0.0 s
Pause after meas. 42	0.0 s
Pause after meas. 43	0.0 s
Pause after meas. 44	0.0 s
Pause after meas. 45	0.0 s
Pause after meas. 46	0.0 s
Pause after meas. 47	0.0 s
Pause after meas. 48	0.0 s
Pause after meas. 49	0.0 s
Pause after meas. 50	0.0 s
Pause after meas. 51	0.0 s
Pause after meas. 52	0.0 s
Pause after meas. 53	0.0 s
Pause after meas. 54	0.0 s
Pause after meas. 55	0.0 s
Pause after meas. 56	0.0 s
Pause after meas. 57	0.0 s
Pause after meas. 58	0.0 s
Pause after meas. 59	0.0 s

Inline - Composing

Inline Composing	Off
Distortion Corr.	Off

Inline - MapIt

Save original images	On
MapIt	None
Flip angle	15.0 deg
Measurements	60
Contrasts	1
TR	5.43 ms
TE	1.87 ms

Sequence - Part 1

Introduction	Off
Dimension	3D
Elliptical scanning	On
Asymmetric echo	Weak
Contrasts	1
Optimization	Min. TE
Multi-slice mode	Sequential
Bandwidth	260 Hz/Px

Sequence - Part 2

RF pulse type	Normal
Gradient mode	Fast
Excitation	Slab-sel.
RF spoiling	On
Incr. Gradient spoiling	Off

Sequence - Assistant

Mode	Off
Allowed delay	0 s
